# Stepwise Structural Simplification of the Dihydroxyanthraquinone Moiety of a Multitarget Rhein-Based Anti-Alzheimer Lead to Improve Drug Metabolism and Pharmacokinetic Properties

**DOI:** 10.3390/pharmaceutics16080982

**Published:** 2024-07-25

**Authors:** Caterina Pont, Anna Sampietro, F. Javier Pérez-Areales, Nunzia Cristiano, Agustí Albalat, Belén Pérez, Manuela Bartolini, Angela De Simone, Vincenza Andrisano, Marta Barenys, Elisabet Teixidó, Raimon Sabaté, M. Isabel Loza, José Brea, Diego Muñoz-Torrero

**Affiliations:** 1 Laboratory of Medicinal Chemistry (CSIC Associated Unit), Faculty of Pharmacy and Food Sciences, University of Barcelona, Av. Joan XXIII 27-31, E-08028 Barcelona, Spain; aitak1989@gmail.com (C.P.); annasampietro@ub.edu (A.S.); fjperezareales@ub.edu (F.J.P.-A.); nunzia.cristiano1991@gmail.com (N.C.); agusalbalat@gmail.com (A.A.); 2 Institute of Biomedicine of the University of Barcelona (IBUB), E-08028 Barcelona, Spain; 3 Department of Pharmacology, Therapeutics and Toxicology, Autonomous University of Barcelona, E-08193 Bellaterra, Spain; belen.perez@uab.cat; 4 Department of Pharmacy and Biotechnology, University of Bologna, Via Belmeloro, 6, I-40126 Bologna, Italy; manuela.bartolini3@unibo.it; 5 Department of Drug Science and Technology, University of Turin, I-10125 Torino, Italy; angela.desimone@unito.it; 6 Department for Life Quality Studies, Alma Mater Studiorum University of Bologna, Corso d’Augusto 237, I-47921 Rimini, Italy; vincenza.andrisano@unibo.it; 7 Toxicology Unit, Department of Pharmacology, Toxicology and Therapeutic Chemistry, Faculty of Pharmacy and Food Sciences, University of Barcelona, Av. Joan XXIII 27-31, E-08028 Barcelona, Spain; mbarenys@ub.edu (M.B.); eteixido1511@ub.edu (E.T.); 8 German Centre for the Protection of Laboratory Animals (Bf3R), German Federal Institute for Risk Assessment (BfR), 10589 Berlin, Germany; 9 Institute of Nutrition and Food Safety of the University of Barcelona (INSA-UB), E-08921 Santa Coloma de Gramenet, Spain; 10 Department of Pharmacy and Pharmaceutical Technology and Physical-Chemistry, Faculty of Pharmacy and Food Sciences, University of Barcelona, Av. Joan XXIII 27-31, E-08028 Barcelona, Spain; rsabate@ub.edu; 11 BioFarma Research Group, Centro Singular de Investigación en Medicina Molecular y Enfermedades Crónicas (CIMUS), Departamento de Farmacología, Farmacia y Tecnología Farmacéutica, Universidade de Santiago de Compostela, Av. de Barcelona s/n, E-15782 Santiago de Compostela, Spain; mabel.loza@usc.es (M.I.L.); pepo.brea@usc.es (J.B.)

**Keywords:** multitarget drugs, Alzheimer’s disease, β-amyloid anti-aggregating agents, tau anti-aggregating agents, BACE-1 inhibitors, acetylcholinesterase inhibitors, butyrylcholinesterase inhibitors, zebrafish embryos, DMPK properties

## Abstract

Multitarget compounds have emerged as promising drug candidates to cope with complex multifactorial diseases, like Alzheimer’s disease (AD). Most multitarget compounds are designed by linking two pharmacophores through a tether chain (linked hybrids), which results in rather large molecules that are particularly useful to hit targets with large binding cavities, but at the expense of suffering from suboptimal physicochemical/pharmacokinetic properties. Molecular size reduction by removal of superfluous structural elements while retaining the key pharmacophoric motifs may represent a compromise solution to achieve both multitargeting and favorable physicochemical/PK properties. Here, we report the stepwise structural simplification of the dihydroxyanthraquinone moiety of a rhein–huprine hybrid lead by hydroxy group removal—ring contraction—ring opening—ring removal, which has led to new analogs that retain or surpass the potency of the lead on its multiple AD targets while exhibiting more favorable drug metabolism and pharmacokinetic (DMPK) properties and safety profile. In particular, the most simplified acetophenone analog displays dual nanomolar inhibition of human acetylcholinesterase and butyrylcholinesterase (IC_50_ = 6 nM and 13 nM, respectively), moderately potent inhibition of human BACE-1 (48% inhibition at 15 µM) and Aβ42 and tau aggregation (73% and 68% inhibition, respectively, at 10 µM), favorable in vitro brain permeation, higher aqueous solubility (18 µM) and plasma stability (100/96/86% remaining in human/mouse/rat plasma after 6 h incubation), and lower acute toxicity in a model organism (zebrafish embryos; LC_50_ >> 100 µM) than the initial lead, thereby confirming the successful lead optimization by structural simplification.

## 1. Introduction

Molecular size is a key determinant of the physicochemical properties of drug candidates and hence, of their likelihood of becoming approved drugs. Relatively large molecules are more prone to suffering from poor physicochemical properties than smaller molecules [1]. However, when it comes to hitting biological targets that have large cavities, with main and secondary binding sites, the situation may be reversed, with large molecules being advantageous, as they can better span those cavities and achieve higher affinity [2]. In those cases, a compromise solution to the dilemma of achieving high affinity or favorable physicochemical properties might be simplifying the chemical structure of a large-molecule hit or lead compound to decrease its molecular size, retaining the key pharmacophore moieties that ensure the desired whole-cavity interactions with the target and removing superfluous structural elements. Here, we report the structural simplification of a lead compound with activity against multiple biological targets for which large ligands may be advantageous, namely acetylcholinesterase (AChE), BACE-1, and the aggregation of β-amyloid peptide (Aβ) and tau protein, developed as a drug candidate against Alzheimer’s disease (AD).

Being the most prevalent neurodegenerative disease and one of the major causes of death worldwide and lacking an effective, safe, and affordable treatment, AD is one of our most urgent unmet medical needs [3,4]. Only four drugs (donepezil, rivastigmine, galantamine, and memantine), which temporarily address the symptoms of the disease, are globally approved [5]. Recently, two eagerly awaited disease-modifying drugs [6] were approved by the FDA, i.e., the Aβ-directed monoclonal antibodies aducanumab (approved in 2021) [7] and lecanemab (approved in 2023) [8]. However, the approval of aducanumab was surrounded by much controversy around its actual efficacy and safety. As a matter of fact, aducanumab was not approved by EMA [9] and was later withdrawn from the market in the USA by the developer Biogen, apparently to be replaced by its more effective successor lecanemab, co-developed by Biogen and Eisai [10]. Nevertheless, the efficacy of lecanemab is still modest, it needs to be administered via infusion, there are some safety concerns related to changes in brain structure and swelling [11,12,13], and its exceedingly high price (26,500 US$/patient/year) may not be affordable for all patients and health systems, generating inequalities in patient access [14]. Thus, there is a dire need to discover new affordable and effective drugs and new ways to address the underlying mechanisms of AD, so that AD progression can be halted or slowed down [15,16]. In this light, targeting multiple complementary disease mechanisms within the complex pathological network of AD [17] seems to be necessary to reach such a goal [18,19].

**Figure 1:**
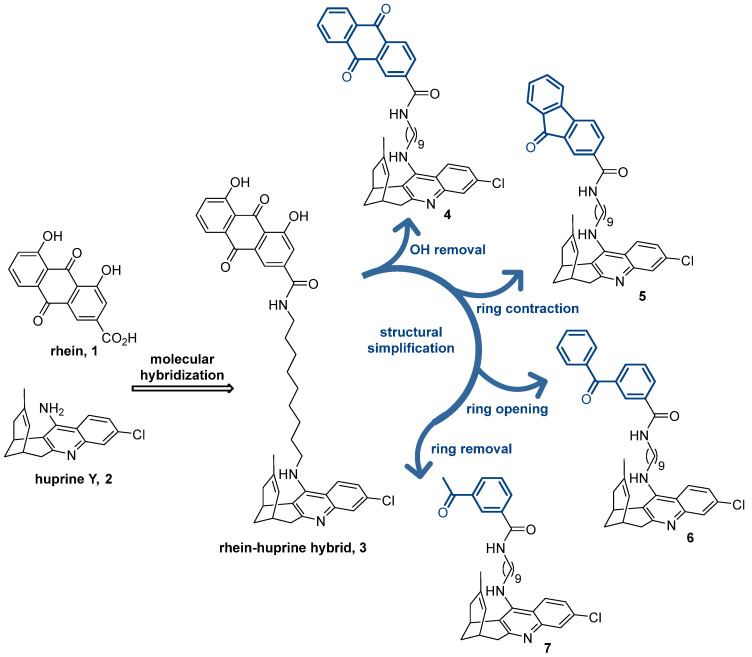
Chemical structures of rhein (**1**), huprine Y (**2**), rhein–huprine hybrid lead (**3**), and design of new hybrids **4**–**7** by stepwise structural simplification of **3**.

Beyond the classical approach of using drug combination therapies, there is very intensive research on new drugs that can hit multiple biological targets within the AD pathological network, i.e., multitarget drugs [20,21,22,23,24,25,26,27]. In most cases, multitarget drugs are designed by the framework combination approach [28], by which the pharmacophores for the modulation of two or more targets are put together into a single hybrid molecule either by fusing or merging the starting scaffolds or, more commonly, by linking them through a tether chain [29]. Very often, the latter hybrids are relatively large molecules, very convenient for multisite binding along the large cavities of some biological targets but not so convenient in terms of physicochemical properties. Some years ago, we developed one of this class of multitarget anti-AD conjugate hybrids, designed by the combination of rhein **1** (Figure 1), a hydroxyanthraquinone scaffold with putative tau anti-aggregating activity [30,31], and the potent AChE inhibitor huprine Y **2** [32,33], or other modified huprine derivatives [34], connected by oligomethylene chains of different lengths. The lead compound **3** features a nonamethylene chain and a huprine Y unit as the optimal linker and AChE inhibitor pharmacophore, respectively (Figure 1) [35]. This large molecule is well accommodated in the large active site cavities of AChE and BACE-1 [36], two prime targets in AD drug discovery due to their role in the cholinergic and amyloid pathologies of the disease. The huprine moiety of **3**, protonated at physiological pH, interacts with the catalytic sites of both AChE and BACE-1, mainly through π-π/cation-π, and ionic interactions, respectively, whereas the rhein dihydroxyanthraquinone scaffold interacts with a peripheral site of AChE (π-π interactions) and with a secondary cryptic pocket of BACE-1 (H-bond). The lead **3** also inhibits the aggregation of Aβ and tau [35], two amyloidogenic proteins with a key pathogenic role in AD [37]. For the latter activities, the molecular size of **3** also seems to play a role, since we have consistently found that conjugate hybrids bearing two pharmacophores with extended π-systems, when connected by linkers of sufficient length, inhibit Aβ and tau aggregation [38].

Within the complex tricyclic core of the dihydroxyanthraquinone moiety of the lead **3**, only the carbonyl group of the most hydrophobic edge (position 9) seems to be involved in H-bond interactions with the secondary site of BACE-1 (with the guanidinium group of Arg307) [36]. Furthermore, the presence of just one of its benzene rings should be enough to enable interactions with the peripheral aromatic site of AChE and the Aβ and tau anti-aggregating activity. Herein, we show the stepwise simplification of the complex dihydroxyanthraquinone moiety of the lead **3** (Figure 1), from the removal of the hydroxy groups (anthraquinone **4**) to ring contraction (fluorenone **5**) and ring opening (benzophenone **6**), up to a minimum, with a final ring removal (acetophenone **7**), to retain the activities regarding AChE, BACE-1, and Aβ and tau aggregation, while affording more favorable physicochemical properties. We report the synthesis, chemical characterization, and consistent biological evaluation of the simplified hybrids **4**–**7**, including (i) multitarget activity profile: in vitro inhibitory activities towards human AChE, human butyrylcholinesterase (BChE), which is another important target for AD treatment, human BACE-1, and Aβ and tau aggregation in a simplified in vivo model using *Escherichia coli* cells genetically modified to express these amyloidogenic proteins; (ii) experimental drug metabolism and pharmacokinetic (DMPK) properties: aqueous solubility, plasma stability, and in vitro brain permeation; and (iii) safety profile: acute toxicity in zebrafish embryos.

## 2. Materials and Methods

### 2.1. Chemistry

#### 2.1.1. General Information

All reagents and solvents were purchased from commercial suppliers unless otherwise stated and used without further purification. The progress of the reactions and column chromatography purifications was monitored by thin-layer chromatography (TLC): TLC plates (aluminum-backed plates with silica gel 60 F_254_ (Merck, ref 1.05554, Darmstadt, Germany)) were developed using CH_2_Cl_2_/MeOH/50% aq. NH_4_OH or hexane/EtOAc/Et_3_N solvent mixtures, and the spots were visualized with UV light and/or 1% aq. KMnO_4_, followed by charring with a heat gun. Column chromatography purifications were performed using silica gel 60 AC.C (35–70 mesh, Carlo Erba, ref 2000027, Milan, Italy). Melting points (mp) were measured in open capillary tubes using an MFB 59510M Gallenkamp apparatus (Loughborough, UK). IR spectra were recorded on an FTIR Perkin-Elmer Spectrum RX I spectrophotometer (Waltham, MA, USA), using the attenuated total reflectance (ATR) technique; significant absorption peaks are given as wavenumbers (cm^−1^). NMR spectra (400 MHz ^1^H/100.6 MHz ^13^C) were recorded on a Mercury-400 spectrometer at the Unitat d’RMN of the Centres Científics i Tecnològics de la Universitat de Barcelona (CCiTUB): chemical shifts are reported in ppm (δ scale) relative to solvent peak (CD_3_OD at 3.31 and 49.0 ppm, in the ^1^H and ^13^C NMR spectra, respectively) and coupling constants in Hertz (Hz); multiplicities are reported as singlet (s), doublet (d), triplet (t), quadruplet (q), multiplet (m), and combinations thereof; assignments are supported by DEPT and ^1^H/^13^C HSQC experiments. For the assignment of the hydrogen atoms at position 13 of the huprine moiety, a *syn* (*anti*) notation is used to denote that these protons are on the same (different) side of the quinoline moiety relative to the cyclohexene ring. High-resolution mass spectra (HRMS, ESI technique) were recorded on a Hewlett-Packard 5988A LC/MSD-TOF instrument (Palo Alto, CA, USA) at the Unitat d’Espectrometria de Masses of CCiTUB. The analytical samples of all the compounds subjected to biological evaluation were previously dried at 45 °C/2 Torr for at least 72 h, using phosphorous pentoxide (P_2_O_5_, Merck, ref 79609, Darmstadt, Germany) and paraffin wax (mp 53–58 °C, Merck, ref 327204, Darmstadt, Germany). The purity of the tested compounds **4**–**7** was in the range of 92–96%, as determined by analytical RP-HPLC (Agilent 1260 Infinity II, coupled to a photodiode array, Santa Clara, CA, USA): column Poroshell 120 EC-C18 (50 × 4.6 mm, 2.7 µm); 40 °C; as mobile phase mixtures of A (0.05% formic acid in water) and B (0.05% formic acid in acetonitrile) (gradient A/B 95:5, 3 min; B, 4 min; A/B 5/95, 1 min); flow rate 0.6 mL/min; and detection wavelength λ = 254 nm.

#### 2.1.2. Synthesis of Compounds **4**–**7**

**Scheme 1:**
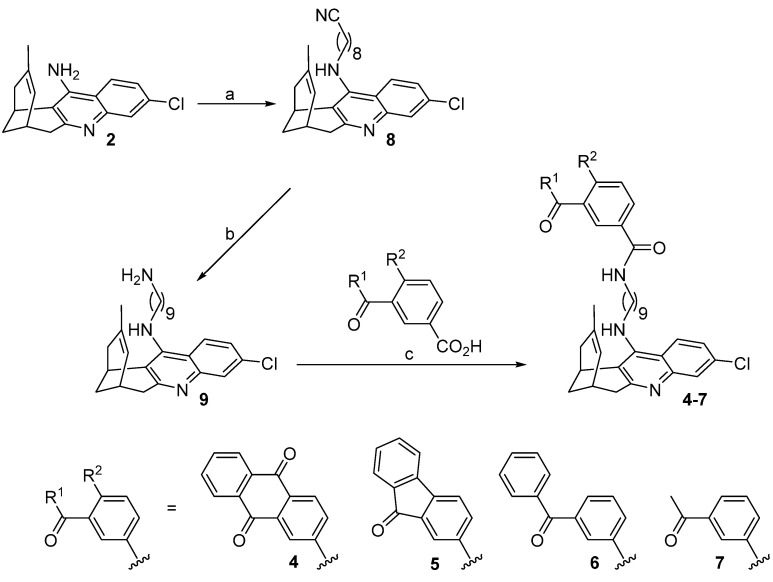
Synthesis of target compounds **4**–**7**. Reagents and conditions: (a) (1) KOH, DMSO, 4 Å molecular sieves, heat gun every 10 min for 1 h, room temperature for 1 h; (2) 9-bromononanenitrile, DMSO, room temperature, overnight, 73%; (b) 4 M LiAlH_4_ in Et_2_O at 0 °C, room temperature, overnight, 96%; (c) carboxylic acid, EDC·HCl, HOBt, Et_3_N, 10:1 EtOAc/DMF, then amine **9**, 10:1 EtOAc/DMF, room temperature, overnight, quantitative (**4**), 70% (**5**), 96% (**6**), quantitative (**7**).

Hybrids **4**–**7** were synthesized as depicted in Scheme 1.

9-[(3-Chloro-6,7,10,11-tetrahydro-9-methyl-7,11-methanocycloocta[*b*]quinolin-12-yl)amino]nonanenenitrile (**8**) [35]

Huprine Y (903 mg, 3.17 mmol), 85% purity KOH (587 mg, 8.89 mmol), and 4Å molecular sieves were mixed in anhydrous DMSO (13 mL). This suspension was stirred while heating for a period of 1 h with the help of a heat gun. Afterwards, the mixture was stirred at room temperature for 1 h. The resulting suspension was treated with 9-bromononanenitrile (761 mg, 3.49 mmol) dissolved in anhydrous DMSO (3 mL), stirred at room temperature overnight, and then 100 mL of 5N NaOH were added and the mixture was extracted with three portions of 70 mL of EtOAc. The organic extracts were washed with four portions of 100 mL of H_2_O and 100 mL of brine, dried (Na_2_SO_4_), and evaporated in vacuo, affording a brown oily crude (2.68 g). Silica gel column chromatography purification of this crude, using hexane/EtOAc/Et_3_N mixtures, from 100:0:0.2 to 87:13:0.2, as eluent, provided nitrile **8** (601 mg, 73% yield) as a yellow oil; *R_f_ =* 0.71 (silica gel, CH_2_Cl_2_/MeOH/50% aq. NH_4_OH 9.8:0.2:0.04); ^1^H NMR (400 MHz, CD_3_OD) *δ*: 1.33–1.47 (m, 8H, 4-H_2_, 5-H_2_, 6-H_2_, 7-H_2_), 1.53 (s, 3H, 9′-CH_3_), 1.60 (tt, *J* = *J*′ = 7.2 Hz, 2H, 3-H_2_), 1.72 (tt, *J* = *J*′ = 7.2 Hz, 2H, 8-H_2_), 1.87 (br d, *J* = 17.2 Hz, H, 10′-H*_endo_*), 1.93 (dm, *J* = 12.4 Hz, 1H, 13′-H*_syn_*), 2.06 (dm, *J* = 12.4 Hz, 1H, 13′-H*_anti_*), 2.40 (t, *J* = 7.2 Hz, 2H, 2-H_2_), 2.55 (dd, *J* = 17.2 Hz, *J*′ = 5.6 Hz, 1H, 10′-H*_exo_*), 2.70 (m, 1H, 7′-H), 2.89 (dt, *J* = 17.6 Hz, *J*′ = 2.0 Hz, 1H, 6′-H*_endo_*), 3.09 (dd, *J* = 17.6 Hz, *J*′ = 5.6 Hz, 1H, 6′-H*_exo_*), 3.45 (m, 1H, 11′-H), 3.58 (td, *J* = 7.2 Hz, *J*′ = 1.6 Hz, 2H, 9-H_2_), 4.85 (s, NH), 5.55 (br d, *J* = 5.6 Hz, 1H, 8′-H), 7.32 (dd, *J* = 9.2 Hz, *J*′ = 2.0 Hz, 1H, 2′-H), 7.73 (d, *J* = 2.0 Hz, 1H, 4′-H), 8.08 (d, *J* = 9.2 Hz, 1H, 1′-H).

*N*-(3-Chloro-6,7,10,11-tetrahydro-9-methyl-7,11-methanocycloocta[*b*]quinolin-12-yl)-1,9-diaminononane (**9**) [35]

A solution of nitrile **8** (1.36 g, 3.22 mmol) in dry Et_2_O (48 mL) was cooled down to 0 °C in an ice bath and treated, dropwise, with a 4 M solution of LiAlH_4_ in Et_2_O (2.40 mL, 9.67 mmol) and the resulting yellow suspension was stirred at room temperature overnight. To the mixture, 60 mL of 1 N NaOH (60 mL) and 120 mL of water were successively added dropwise, and it was extracted with three portions of 120 mL of EtOAc. The organic layers were dried (Na_2_SO_4_) and evaporated in vacuo, providing the desired amine **9** (1.30 g, 96% yield) in the form of yellow oil; *R_f_ =* 0.53 (silica gel, CH_2_Cl_2_/MeOH/50% aq. NH_4_OH 9.8:0.2:0.07); ^1^H NMR (400 MHz, CD_3_OD) *δ*: 1.27–1.37 (m, 10H, 3-H_2_, 4-H_2_, 5-H_2_, 6-H_2_, 7-H_2_), 1.43 (tt, *J* = *J*′ = 6.8 Hz, 2H, 8-H_2_), 1.53 (s, 3H, 9′-CH_3_), 1.71 (tt, *J* = *J*′ = 7.2 Hz, 2H, 2-H_2_), 1.87 (br d, *J* = 17.2 Hz, 1H, 10′-H*_endo_*), 1.93 (dm, *J* = 12.4 Hz, 1H, 13′-H*_syn_*), 2.07 (dm, *J* = 12.4 Hz, 1H, 13′-H*_anti_*), 2.54 (dd, *J* = 17.2 Hz, *J*′ = 5.2 Hz, 1H, 10′-H*_exo_*), 2.61 (t, *J* = 7.2 Hz, 2H, 9-H_2_), 2.70 (m, 1H, 7′-H), 2.89 (dt, *J* = 17.2 Hz, *J*′ = 2.0 Hz, 1H, 6′-H*_endo_*), 3.09 (dd, *J* = 17.2 Hz, *J*′ = 5.6 Hz, 1H, 6′-H*_exo_*), 3.45 (m, 1H, 11′-H), 3.57 (td, *J* = 7.2 Hz, *J*′ = 2.4 Hz, 2H, 1-H_2_), 4.85 (s, NH, NH_2_), 5.55 (br d, *J* = 4.8 Hz, 1H, 8′-H), 7.31 (dd, *J* = 8.8 Hz, *J*′ = 2.0 Hz, 1H, 2′-H), 7.73 (d, *J* = 2.0 Hz, 1H, 4′-H), 8.08 (d, *J* = 8.8 Hz, 1H, 1′-H).

*N*-{9-[(3-Chloro-6,7,10,11-tetrahydro-9-methyl-7,11-methanocycloocta[*b*]quinolin-12-yl)amino]nonyl}anthraquinone-2-carboxamide (**4**)

To a suspension of anthraquinone-2-carboxylic acid (56 mg, 0.22 mmol) in a 10:1 mixture of EtOAc/DMF (4.4 mL), 1-ethyl-3-(3-dimethylaminopropyl)carbodiimide hydrochloride (EDC·HCl, 58 mg, 0.30 mmol), Et_3_N (0.07 mL, 0.50 mmol), and 1-hydroxybenzotriazole (HOBt, 41 mg, 0.30 mmol) were added. After stirring for 10 min at room temperature, a solution of amine **9** (85 mg, 0.20 mmol) in 10:1 EtOAc/DMF (2.2 mL) was added and the reaction mixture was stirred at room temperature overnight. Thereafter, it was evaporated to dryness and the resulting yellow oil (307 mg) was purified through silica gel column chromatography (hexane/EtOAc/Et_3_N mixtures in the ratio 100:0:0.2 to 75:25:0.5). On elution with hexane/EtOAc/Et_3_N 80:20:0.2, the hybrid **4** (187 mg, quantitative yield) was obtained as a yellow oil; *R_f_ =* 0.22 (silica gel, hexane/EtOAc/Et_3_N 6:4:0.02).

The analytical sample of **4**, in the form of the hydrochloride salt (**4**·HCl), was prepared by treatment of a 0.2 μm PTFE-filtered solution of the free base isolated after column chromatography in CH_2_Cl_2_ (2 mL) with an Et_2_O solution of HCl (1 mL, 1.17 M), followed by evaporation to dryness and washing of the resulting solid with pentane (2 × 2 mL). After drying the solid at 45 °C/2 Torr for 5 days, **4**·HCl (94 mg) was obtained as a yellowish solid; mp: 123–125 °C; IR (ATR) *ν*: 3600–2200 (max at 3249, 3064, 2926, 2855), 1674, 1633, 1584, 1454, 1326, 1281, 1253, 931, 707 cm^−1^; ^1^H NMR (400 MHz, CD_3_OD) *δ*: 1.37–1.50 (m, 10H, 3′-H_2_, 4′-H_2_, 5′-H_2_, 6′-H_2_, 7′-H_2_), 1.59 (s, 3H, 9″-CH_3_), 1.68 (tt, *J* = *J*′ = 7.2 Hz, 2H, 2′-H_2_), 1.85 (m, 2H, 8′-H_2_), superimposed in part 1.89–1.96 (m, 2H, 10″-H*_endo_*, 13″-H*_syn_*), 2.07 (dm, *J* = 11.2 Hz, 1H, 13″-H*_anti_*), 2.53 (dd, *J* = 18.0 Hz, *J*′ = 5.2 Hz, 1H, 10″-H*_exo_*), 2.75 (m, 1H, 7″-H), superimposed in part 2.80 (dt, *J* = 18.0 Hz, *J*′ = 2.0 Hz, 1H, 6″-H*_endo_*), 3.15 (dd, *J* = 18.0 Hz, *J*′ = 5.2 Hz, 1H, 6″-H*_exo_*), 3.38 (m, 1H, 11″-H), 3.44 (td, *J* = 7.2 Hz, *J*′ = 2.4 Hz, 2H, 1′-H_2_), 3.89 (td, *J* = 7.6 Hz, *J*′ = 4.4 Hz, 2H, 9′-H_2_), 4.85 (s, NH, ^+^NH), 5.58 (br d, *J* = 5.6 Hz, 1H, 8″-H), 7.43 (dd, *J* = 9.2 Hz, *J*′ = 2.4 Hz, 1H, 2″-H), 7.59 (d, *J* = 2.4 Hz, 1H, 4″-H), 7.87 (ddd, *J* = *J*′ = 7.2 Hz, *J*″ = 2.0 Hz, 1H), 7.90 (ddd, *J* = *J*′ = 7.2 Hz, *J*″ = 2.0 Hz, 1H) (6-H, 7-H), 8.19–8.28 (m, 4H, 3-H, 4-H, 5-H, 8-H), 8.31 (d, *J* = 9.2 Hz, 1H, 1″-H), 8.63 (d, *J* = 2.0 Hz, 1H, 1-H); ^13^C NMR (100.6 MHz, CD_3_OD) *δ*: 23.5 (CH_3_, 9″-CH_3_), 27.2 (CH, C11″), 27.7 (2CH_2_, C6′, C7′), 27.8 (CH, C7″), 29.3 (CH_2_, C13″), 29.9 (2CH_2_), 30.1 (CH_2_), 30.2 (CH_2_) (C2′, C3′, C4′, C5′), 31.2 (CH_2_, C8′), 36.0 (2CH_2_, C6″, C10″), 41.0 (CH_2_, C1′), 49.6 (CH_2_, C9′), 115.4 (C, C12a″), 117.5 (C, C11a″), 119.1 (CH, C4″), 125.1 (CH, C8″), 126.6 (CH, C2″), 126.8 (CH, C1), 128.0 (CH), 128.1 (CH), 128.4 (CH) (C4, C5, C8), 129.3 (CH, C1″), 133.6 (CH, C3), 134.55 (C), 134.56 (C), 134.59 (C), 134.7 (C) (C4a, C8a, C10a, C9″), 135.59 (CH), 135.65 (CH) (C6, C7), 136.2 (C, C9a), 140.2 (C, C3″), 140.8 (C, C4a″), 141.1 (C, C2), 151.0 (C, C5a″), 156.7 (C, C12″), 168.1 (C, CONH), 183.36 (C), 183.38 (C) (C9, C10); HRMS ESI: calculated for [C_41_H_42_^35^ClN_3_O_3_ + H]^+^ 660.2987; found 660.2983; HPLC purity: 93%.

*N*-{9-[(3-Chloro-6,7,10,11-tetrahydro-9-methyl-7,11-methanocycloocta[*b*]quinolin-12-yl)amino]nonyl}-9-fluorenone-2-carboxamide (**5**)

This compound was prepared following the procedure described for **4**. From a suspension of 9-fluorenone-2-carboxylic acid (70 mg, 0.31 mmol), EDC·HCl (66 mg, 0.42 mmol), Et_3_N (0.08 mL, 0.56 mmol), and HOBt (58 mg, 0.42 mmol) in 10:1 EtOAc/DMF (3.8 mL) and a solution of amine **9** (120 mg, 0.28 mmol) in 10:1 EtOAc/DMF (5.5 mL), a yellow oil (449 mg) was obtained and subjected to silica gel column chromatography purification (CH_2_Cl_2_/MeOH/50% aq. NH_4_OH mixtures in the ratio 100:0:0.4 to 99.9:0.1:0.4). Hybrid **5** (124 mg, 70% yield) was obtained as a yellow oil; *R_f_ =* 0.83 (silica gel, CH_2_Cl_2_/MeOH/50% aq. NH_4_OH 9:1:0.1).

The analytical sample of **5**·HCl was prepared as described before, from a solution of the free base of **5** in CH_2_Cl_2_ (4 mL) and an Et_2_O solution of HCl (2 mL, 1.17 M). After washing with pentane (3 × 4 mL), the resulting brown solid was taken up in a few drops of MeOH, precipitated after dropwise addition of EtOAc, and dried at 45 °C/2 Torr for 3 days. Then, **5**·HCl (88 mg) was obtained as a brown solid: mp: 217–219 °C; IR (ATR) *ν*: 3600–2300 (max at 3261, 3056, 2926, 2854), 1715, 1631, 1582, 1511, 1456, 1360, 1307, 1265, 1098, 1041, 1021, 928, 914, 737, 722, 660 cm^−1^; ^1^H NMR (400 MHz, CD_3_OD) *δ*: 1.35–1.50 (m, 10H, 3′-H_2_, 4′-H_2_, 5′-H_2_, 6′-H_2_, 7′-CH_2_), 1.59 (s, 3H, 9″-CH_3_), 1.64 (tt, *J* = *J*′ = 6.8 Hz, 2H, 2′-H_2_), 1.84 (tt, *J* = *J*′ = 7.2 Hz, 2H, 8′-H_2_), superimposed in part 1.92 (dm, *J* = 12.4 Hz, 1H, 13″-H*_syn_*), 1.93 (dm, *J* = 17.6 Hz, 1H, 10″-H*_endo_*), 2.06 (dm, *J* = 12.4 Hz, 1H, 13″-H*_anti_*), 2.53 (dd, *J* = 17.6 Hz, *J*′ = 5.2 Hz, 1H, 10″-H*_exo_*), 2.75 (m, 1H, 7″-H), 2.82 (br d, *J* = 17.2 Hz, 1H, 6″-H*_endo_*), 3.16 (dd, *J* = 17.2 Hz, *J*′ = 5.6 Hz, 1H, 6″-H*_exo_*), 3.35–3.45 (m, 2H, 1′-H_2_, 11″-H), 3.89 (m, 2H, 9′-H_2_), 4.85 (s, NH, ^+^NH), 5.58 (br d, *J* = 5.6 Hz, 1H, 8″-H), 7.38 (ddd, *J* = *J*′ = 7.2 Hz, *J*″ = 1.2 Hz, 1H, 7-H), 7.45 (dd, *J* = 9.2 Hz, *J*′ = 2.0 Hz, 1H, 2″-H), 7.56 (br d, *J* = 7.2 Hz, 1H, 8-H), superimposed in part 7.58 (ddd, *J* = *J*′ = 7.2 Hz, *J*″ = 1.2 Hz, 1H, 6-H), 7.63 (d, *J* = 2.0 Hz, 1H, 4″-H), 7.67 (br d, *J* = 7.2 Hz, 1H, 5-H), 7.70 (d, *J* = 7.6 Hz, 1H, 4-H), 7.97 (d, *J* = 2.0 Hz, 1H, 1-H), 8.01 (dd, *J* = 7.6 Hz, *J*′ = 2.0 Hz, 1H, 3-H), 8.26 (d, *J* = 9.2 Hz, 1H, 1″-H); ^13^C NMR (100.6 MHz, CD_3_OD) *δ*: 23.5 (CH_3_, 9″-CH_3_), 27.3 (CH, C11″), 27.6 (2CH_2_, C6′, C7′), 27.8 (CH, C7″), 29.3 (CH_2_, C13″), 29.9 (2CH_2_), 30.2 (2CH_2_) (C2′, C3′, C4′, C5′), 31.2 (CH_2_, C8′), 36.0 (2CH_2_, C6″, C10″), 40.9 (CH_2_, C1′), 49.6 (CH_2_, C9′), 115.5 (C, C12a″), 117.5 (C, C11a″), 119.2 (CH, C4″), 121.8 (CH, C4), 122.5 (CH, C5), 123.5 (CH, C1), 125.09 (CH, C8), 125.14 (CH, C8″), 126.6 (CH, C2″), 129.3 (CH, C1″), 131.1 (CH, C7), 134.6 (C, C9″), 135.3 (C, C4b), 135.4 (CH, C3), 135.5 (CH, C6), 136.5 (C), 136.9 (C) (C8a, C4a), 140.2 (C, C3″), 140.9 (C, C4a″), 144.7 (C, C2), 148.3 (C, C9a), 151.1 (C, C5a″), 156.7 (C, C12″), 168.7 (C, CONH), 185.1 (C, C9); HRMS ESI: calculated for [C_40_H_42_^35^ClN_3_O_2_ + H]^+^ 632.3038; found: 632.3034; HPLC purity: 92%.

3-Benzoyl-*N*-{9-[(3-chloro-6,7,10,11-tetrahydro-9-methyl-7,11-methanocycloocta[*b*]quinolin-12-yl)amino]nonyl}benzamide (**6**)

This compound was prepared following the procedure described for **4**. From a suspension of 3-benzoylbenzoic acid (88 mg, 0.39 mmol), EDC·HCl (82 mg, 0.53 mmol), Et_3_N (0.1 mL, 0.72 mmol), and HOBt (72 mg, 0.53 mmol) in 10:1 EtOAc/DMF (4.5 mL) and a solution of amine **9** (150 mg, 0.35 mmol) in 10:1 EtOAc/DMF (10.6 mL), a yellow oil (629 mg) was obtained and subjected to silica gel column chromatography purification (CH_2_Cl_2_/MeOH/50% aq. NH_4_OH mixtures in the ratio 100:0:0.4 to 99.6:0.4:0.4). Hybrid **6** (216 mg, 96% yield) was obtained as a yellow oil; *R_f_ =* 0.84 (silica gel, CH_2_Cl_2_/MeOH/50% aq. NH_4_OH 9:1:0.1).

The analytical sample of **6**·HCl was prepared as described before, from a solution of the free base of **6** in CH_2_Cl_2_ (5 mL) and an Et_2_O solution of HCl (2 mL, 1.17 M). After washing with pentane (3 × 4 mL), the resulting brown solid was taken up in a few drops of MeOH, precipitated after dropwise addition of EtOAc, and dried at 45 °C/2 Torr for 3 days. Then, **6**·HCl (95 mg) was obtained as a brown solid: mp: 124–126 °C; IR (ATR) *ν*: 3600–2400 (max at 3239, 3060, 2926, 2856), 1657, 1632, 1582, 1568, 1525, 1447, 1360, 1307, 1267, 1180, 1090, 930, 822, 768, 713, 697, 639 cm^−1^; ^1^H NMR (400 MHz, CD_3_OD) *δ*: 1.34–1.45 (m, 10H, 3′-H_2_, 4′-H_2_, 5′-H_2_, 6′-H_2_, 7′-H_2_), 1.56 (s, 3H, 9″-CH_3_), superimposed in part 1.60 (m, 2H, 2′-H_2_), 1.84 (tt, *J* = *J*′ = 7.2 Hz, 2H, 8′-H_2_), superimposed in part 1.92 (dm, *J* = 12.0 Hz, 1H, 13″-H*_syn_*), 1.93 (br d, *J* = 17.2 Hz, 1H, 10″-H*_endo_*), 2.06 (dm, *J* = 12.0 Hz, 1H, 13″-H*_anti_*), 2.53 (dd, *J* = 17.2 Hz, *J*′ = 5.6 Hz, 1H, 10″-H*_exo_*), 2.75 (m, 1H, 7″-H), 2.87 (ddd, *J* = 18.0 Hz, *J*′ = *J*″ = 1.6 Hz, 1H, 6″-H*_endo_*), 3.19 (dd, *J* = 18.0 Hz, *J*′ = 5.6 Hz, 1H, 6″-H*_exo_*), 3.37 (t, *J*′ = 7.2 Hz, 2H, 1′-H_2_), 3.43 (m, 1H, 11″-H), 3.96 (td, *J* = 7.2 Hz, *J*′ = 2.8 Hz, 2H, 9′-H_2_), 4.85 (s, NH, ^+^NH), 5.57 (br d, *J* = 5.2 Hz, 1H, 8″-H), 7.50–7.57 (m, 3H, 2″-H, benzoyl H*_meta_*), 7.62 (dd, *J* = *J*′ = 8.0 Hz, 1H, 5-H), 7.65 (tt, *J* = 7.6 Hz, *J*′ = 1.2 Hz, 1H, benzoyl H*_para_*), 7.74–7.77 (m, 3H, 4″-H, benzoyl H*_ortho_*), 7.88 (dt, *J* = 7.6 Hz, *J*′ = 1.6 Hz, 1H), 8.07 (dt, *J* = 8.0 Hz, *J*′ = 1.6 Hz, 1H) (4-H, 6-H), 8.20 (t, *J* = 1.6 Hz, 1H, 2-H), 8.36 (d, *J* = 9.2 Hz, 1H, 1″-H); ^13^C NMR (100.6 MHz, CD_3_OD) *δ*: 23.5 (CH_3_, 9″-CH_3_), 27.3 (CH, C11″), 27.7 (CH_2_), 27.9 (CH_2_) (C6′, C7′), 27.8 (CH, C7″), 29.3 (CH_2_, C13″), 30.0 (CH_2_), 30.1 (CH_2_), 30.3 (CH_2_), 30.4 (CH_2_) (C2′, C3′, C4′, C5′), 31.2 (CH_2_, C8′), 36.0 (CH_2_), 36.1 (CH_2_) (C6″, C10″), 41.0 (CH_2_, C1′), 49.7 (CH_2_, C9′), 115.6 (C, C12a″), 117.6 (C, C11a″), 119.2 (CH, C4″), 125.1 (CH, C8″), 126.6 (CH, C2″), 129.4 (CH, C1″), 129.59 (CH, C5), 129.64 (2CH, benzoyl C*_meta_*), 129.8 (CH, benzoyl C*_para_*), 131.0 (2CH, benzoyl C*_ortho_*), 132.1 (CH), 133.7 (CH), 134.1 (CH) (C2, C4, C6), 134.5 (C, C9″), 136.3 (C), 138.4 (C), 139.2 (C) (C1, C3, benzoyl C*_ipso_*), 140.2 (C, C3″), 141.0 (C, C4a″), 151.2 (C, C5a″), 156.9 (C, C12″), 169.0 (C, CONH), 197.6 (C, benzoyl CO); HRMS ESI: calculated for [C_40_H_44_^35^ClN_3_O_2_ + H]^+^: 634.3195; found 634.3187; HPLC purity: 95%.

3-Acetyl-*N*-{9-[(3-chloro-6,7,10,11-tetrahydro-9-methyl-7,11-methanocycloocta[*b*]quinolin-12-yl)amino]nonyl}benzamide (**7**)

This compound was prepared following the procedure described for **4**. From a suspension of 3-acetylbenzoic acid (36 mg, 0.22 mmol), EDC·HCl (58 mg, 0.30 mmol), Et_3_N (0.07 mL, 0.50 mmol), and HOBt (41 mg, 0.30 mmol) in 10:1 EtOAc/DMF (4.4 mL) and a solution of amine **9** (85 mg, 0.20 mmol) in 10:1 EtOAc/DMF (2.2 mL), a yellow oil (272 mg) was obtained and subjected to silica gel column chromatography purification (hexane/EtOAc/Et_3_N mixtures in the ratio 100:0:0.2 to 60:40:0.5). On elution with hexane/EtOAc/Et_3_N 70:30:0.2, hybrid **7** (146 mg, quantitative yield) was obtained as a yellow oil; *R_f_ =* 0.13 (silica gel, hexane/EtOAc/Et_3_N 6:4:0.02).

The analytical sample of **7**·HCl was prepared as described before, from a solution of the free base of **7** in CH_2_Cl_2_ (2 mL) and an Et_2_O solution of HCl (1 mL, 1.17 M). After washing with pentane (2 × 4 mL) and drying at 45 °C/2 Torr for 5 days, **7**·HCl (92 mg) was obtained as a yellowish solid: mp: 85–87 °C; IR (ATR) *ν*: 3600–2300 (max at 3251, 3056, 2926, 2854), 1684, 1632, 1582, 1568, 1542, 1463, 1455, 1437, 1359, 1328, 1305, 1252, 1090, 930, 914, 819, 768, 755, 690, 590 cm^−1^; ^1^H NMR (400 MHz, CD_3_OD) *δ*: 1.33–1.47 (m, 10H, 3′-H_2_, 4′-H_2_, 5′-H_2_, 6′-H_2_, 7′-H_2_), 1.58 (s, 3H, 9″-CH_3_), superimposed in part 1.63 (tt, *J* = *J*′ = 7.2 Hz, 2H, 2′-H_2_), 1.85 (tt, *J* = *J*′ = 7.2 Hz, 2H, 8′-H_2_), superimposed in part 1.90 (dm, *J* = 12.8 Hz, 1H, 13″-H*_syn_*), 1.93 (dm, *J* = 17.6 Hz, 1H, 10″-H*_endo_*), 2.08 (dm, *J* = 12.8 Hz, 1H, 13″-H*_anti_*), 2.54 (dd, *J* = 17.6 Hz, *J*′ = 5.2 Hz, 1H, 10″-H*_exo_*), 2.64 (s, 3H, CH_3_CO), 2.77 (m, 1H, 7″-H), 2.85 (ddd, *J* = 17.6 Hz, *J*′ = *J*″ = 1.6 Hz, 1H, 6″-H*_endo_*), 3.20 (dd, *J* = 17.6 Hz, *J*′ = 5.6 Hz, 1H, 6″-H*_exo_*), 3.39 (t, *J*′ = 7.2 Hz, 2H, 1′-H_2_), 3.43 (m, 1H, 11″-H), 3.97 (td, *J* = 7.2 Hz, *J*′ = 3.2 Hz, 2H, 9′-H_2_), 4.85 (s, NH, ^+^NH), 5.58 (br d, *J* = 5.6 Hz, 1H, 8″-H), 7.55 (dd, *J* = 9.2 Hz, *J*′ = 2.0 Hz, 1H, 2″-H), 7.60 (ddd, *J* = *J*′ = 8.0 Hz, *J*″ = 0.8 Hz, 1H, 5-H), 7.74 (d, *J* = 2.0 Hz, 1H, 4″-H), 8.04 (ddd, *J* = 7.6 Hz, *J*′ = 1.6 Hz, *J*″ = 1.2 Hz, 1H), 8.14 (ddd, *J* = 7.6 Hz, *J*′ = 1.6 Hz, *J*″ = 1.2 Hz, 1H) (4-H, 6-H), 8.39 (d, *J* = 9.2 Hz, 1H, 1″-H), 8.41 (td, *J* = 1.6 Hz, *J*′ = 0.8 Hz, 1H, 2-H); ^13^C NMR (100.6 MHz, CD_3_OD) *δ*: 23.5 (CH_3_, 9″-CH_3_), 26.8 (CH_3_, *C*H_3_CO), 27.3 (CH, C11″), 27.7 (CH_2_), 27.9 (CH_2_) (C6′, C7′), 27.8 (CH, C7″), 29.3 (CH_2_, C13″), 30.1 (CH_2_), 30.2 (CH_2_), 30.40 (CH_2_), 30.43 (CH_2_) (C2′, C3′, C4′, C5′), 31.2 (CH_2_, C8′), 36.0 (CH_2_), 36.1 (CH_2_) (C6″, C10″), 41.0 (CH_2_, C1′), 49.7 (CH_2_, C9′), 115.6 (C, C12a″), 117.6 (C, C11a″), 119.1 (CH, C4″), 125.1 (CH, C8″), 126.6 (CH, C2″), 128.1 (CH, C5), 129.5 (CH, C1″), 130.0 (CH), 132.2 (CH), 132.8 (CH) (C2, C4, C6), 134.5 (C, C9″), 136.4 (C), 138.5 (C) (C1, C3), 140.2 (C, C3″), 141.0 (C, C4a″), 151.2 (C, C5a″), 156.9 (C, C12″), 169.1 (C, CONH), 199.5 (C, CH_3_*C*O); HRMS ESI: calculated for [C_35_H_42_^35^ClN_3_O_2_ + H]^+^ 572.3038, found 572.3039; HPLC purity: 96%.

### 2.2. Biological Activity Profiling

#### 2.2.1. In Vitro hAChE and hBChE Inhibition

The human recombinant AChE (hAChE) and human serum BChE (hBChE) inhibitory activities of the new compounds **4**–**7** were tested by the widely used method of Ellman [39], using enzymes supplied by Sigma (Milan, Italy). The stock and assay solutions were prepared as follows:

Stock hAChE solution: solution of the lyophilized powder in 0.1% Triton X-100/0.1 M potassium phosphate (pH 8.0).

Stock hBChE solution: solution of the lyophilized powder in 0.1% aq. gelatin.

Stock solutions of the new compounds: 1 mM solutions in methanol, with further dilutions in this solvent to the desired concentrations.

Assay solutions: mixture of 340 μM 5,5′-dithiobis(2-nitrobenzoic acid), hAChE or hBChE (0.02 unit/mL), and 0.1 M potassium phosphate (pH 8.0)

Acetylthiocholine iodide or butyrylthiocholine iodide (550 μM), as substrates, were added to the assay solutions, with or without the compounds to be tested, previously preincubated for 20 min at 37 °C. To correct for the potential non-enzymatic hydrolysis of the substrates, blank solutions, which did not contain any enzyme, were also prepared. A Jasco V-530 double-beam spectrophotometer, featuring cuvette holders kept at 37 °C, was used to monitor the initial rates at 412 nm. To calculate the IC_50_ values for hAChE and hBChE inhibition (Microcal Origin 3.5 software, Microcal Software, Inc., Northhampton, MA, USA), five or more increasing concentrations of the new compounds, which produced inhibition in the range 20–80%, were tested.

#### 2.2.2. In Vitro hBACE-1 Inhibition

The inhibition of human recombinant BACE-1 (hBACE-1) (Invitrogen, Carlsbad, CA, USA) by hybrids **4**–**7** was evaluated by a fluorometric method. The substrate (Panvera peptide, 10 μL, 250 nM final concentration) was added to a solution (10 μL) of hybrids **4**–**7** or buffer in control wells (20 mM sodium acetate at pH 4.5, with 0.1% *w*/*v* CHAPS). After the addition of the enzyme (10 μL, 12.91 mU), the assay solution was incubated for 1 h at 37 °C. Thereafter, STOP solution (10 μL, 2.5 M sodium acetate) was added and the fluorescence was read at λ_em_ = 544 nm and λ_ex_ = 590 nm. The DMSO concentration in the assay solution was kept <5% (*v*/*v*) to ensure no significant loss of BACE-1 activity. The background signal, determined in control wells with all the components of the assay but BACE-1, was subtracted. The fluorescence intensities in the presence of increasing concentrations or in the absence of hybrids **4**–**7** were compared to calculate the percent of enzyme inhibition produced by the compounds, by applying the following expression: 100 − (IF_i_/IF_o_ × 100), in which IF_i_ and IF_o_ are the fluorescence intensities in the presence and in the absence of the compounds, respectively. Additionally, the IC_50_ values were calculated (GraphPad Prism 4.03 software, GraphPad Software Inc., San Diego, CA, USA) and are given as the mean ± SEM of at least two experiments, each performed in triplicate.

#### 2.2.3. Aβ42 and Tau Aggregation Inhibition in Genetically Modified *Escherichia coli* Cells

For Aβ42 overexpression, M9 minimal medium (10 mL) containing kanamycin (50 μg/mL) was added to a colony of *E. coli* BL21 (DE3) competent cells, bearing the pET28a plasmid (Novagen, Inc., Madison, WI, USA), which contains the DNA sequence encoding for Aβ42. After overnight culture, the volume necessary to obtain a 1:500 dilution was added to fresh M9 minimal medium that contained kanamycin (50 μg/mL) and thioflavin-S (Th-S, 250 µM).

For tau overexpression, M9 minimal medium (10 mL) containing glucose (0.5%), ampicillin (50 μg/mL), chloramphenicol (12.5 μg/mL), and *E. coli* BL21 (DE3) cells bearing the pTARA plasmid, which carries the RNA polymerase gene of the T7 phage (T7RP) under control of the PBAD promoter, and subsequent transformation with the pRKT42 vector were used instead. In this case, the overnight culture for a 1:500 dilution was added to fresh M9 minimal medium containing glucose (0.5%), chloramphenicol (12.5 μg/mL), ampicillin (50 μg/mL), and Th-S (250 µM).

For Aβ42 overexpression, the corresponding *E. coli* cell cultures were kept growing overnight (37 °C; 250 rpm) until they reached an OD_600_ = 0.6. These cultures (980 μL) were added to a mixture of 10 μL of a solution of the new compounds in DMSO and 10 μL of 100 mM isopropyl 1-thio-β-D-galactopyranoside (IPTG), to reach a final compound concentration of 10 μM. The same procedure was used for tau overexpression but employing 10 μL of 25% arabinose instead of IPTG. The resulting *E. coli* cultures were kept growing overnight (37 °C; 1400 rpm) using an Eppendorf Thermomixer (Hamburg, Germany). To prepare a negative control for the assay, showing the highest amount of the amyloidogenic protein, the same quantity of DMSO, without the compounds to be tested, was added to the sample. With the double purpose of obtaining positive controls devoid of the amyloidogenic proteins and assessing the potential intrinsic toxicity of the new compounds, we also prepared samples that had not been treated with either IPTG or arabinose (non-induced samples). 

To assess the inhibitory activity of compounds **4**–**7** on Aβ42 and tau aggregation within *E. coli* cells, a fluorescence assay was employed, using Th-S (2500 mM stock solution, T1892, Sigma, St. Louis, MO, USA) in double-distilled water (Milli-Q system, Millipore, Bay City, MI, USA) and an Aminco Bowman Series 2 luminescence spectrophotometer (Aminco-Bowman AB2, SLM Aminco, Rochester, NY, USA), working at 25 °C in the wavelength range of 460–600 nm, with λ_ex_ = 445 nm, slit widths of 4 nm, and λ_em_ = 485 nm. The results are given as the average of nine independent experiments, each performed in triplicate. The known Aβ42 and tau aggregation inhibitor DP128 was used here as a positive control, which showed 78.1 ± 1.7 and 69.9 ± 0.7% inhibition at 10 μM, respectively.

### 2.3. In Vitro DMPK Property Profiling

#### 2.3.1. Aqueous Solubility

In a 384-well Greiner 781801 transparent plate (Kremsmünster, Austria), 10^−2^ M stock solutions of the new compounds were diluted with a mixture of DMSO (1%) and PBS (99%). The resulting solutions were incubated at 37 °C. After 2 h incubation, the samples were read using a BMG LABTECH NEPHELOstar Plus laser nephelometer (Ortenberg, Germany).

The obtained results were adjusted to a segmented regression to determine the maximum concentration at which compounds **4**–**7** were soluble. Progesterone (solubility: described, 3.7 μM [40]; found, 5.9 μM) and digoxin (solubility: described, 59.3 μM [40]; found, 86.3 μM) were used as reference compounds.

#### 2.3.2. Plasma Stability

The plasma stability of the new compounds was tested using in human, mouse, and rat plasma (Seralab). One hundred μL of 5 μM solutions of the new compounds in plasma were plated and incubated at 37 °C for 0, 60, 120, and 360 min, and then treated with 300 μL of acetonitrile and centrifuged (4000× *g*; 60 min; 4 °C). Sample quantification was performed by UPLC/MS/MS analysis of the resulting supernatant using a UPLC QSM Waters Acquity apparatus, equipped with a Waters ACQUITY BEH C18 1.7 μm 2.1 × 50 mm column, eluting with 0.1% formic acid in water or in acetonitrile, with a flow of 0.6 mL/min. The gradient indicated in Table 1 was used in this assay.

Compound concentration was calculated from the MS peak areas and expressed as the percentage of remaining compound at the different tested times.

#### 2.3.3. Brain Permeability

The parallel artificial membrane permeability assay for the blood–brain barrier (PAMPA-BBB) [41] was used to assess the in vitro brain permeability of the new compounds. For the assay, the compounds were dissolved in mixtures of PBS and ethanol (70:30). Fourteen drugs were included for the assay validation (Table 2). The following correlation was obtained: *P*_e_ (exp) = 1.6217 *P*_e_ (lit) − 1.2723 (R^2^ = 0.9362), and the different ranges of BBB permeation were established as follows: high BBB permeation (CNS+), with *P*_e_ (10^−6^ cm/s) > 5.21; low BBB permeation (CNS–), with *P*_e_ (10^−6^ cm/s) < 1.97; and uncertain BBB permeation (CNS±), with 5.21 > *P*_e_ (10^−6^ cm/s) > 1.97. Data are given as the mean ± SD of three independent experiments, each performed in triplicate.

### 2.4. Acute Toxicity in Zebrafish Embryos

The care of adult zebrafish was authorized by the University of Barcelona’s Ethical Committee for Animal Experimentation (CEEA) and received approval from the Department of Environment and Housing of the Generalitat de Catalunya (license number 334/18). All procedures adhered to the regulations outlined in Decree 214/1997 of the Generalitat de Catalunya, which oversees the use of animals in scientific research and experimentation.

This acute toxicity assay was carried out following the OECD Guideline number 236 (Fish Embryo Acute Toxicity (FET) Test [42], with modifications [43]. One to two hours after the spawning of eggs, they were collected and cleaned with OECD water diluted 1:5 as detailed in ISO 7346-1 and 7346-2 [44,45] (2 mM CaCl_2_·2H_2_O; 0.75 mM NaHCO_3_; 0.5 mM MgSO_4_·7H_2_O; and 0.07 mM KCl). Thereafter, some eggs were selected using a dissection stereomicroscope (Motic SMZ168, Motic China Group, LTD., Hong Kong, China). Fertilized, synchronously divided zebrafish embryos were distributed, randomly, in 6-well plates, with ten embryos per well, and were exposed to increasing concentrations of compounds **3**–**7** diluted in Danieau’s solution 0.3X (5 mL/well; 17.4 mM NaCl; 0.23 mM KCl; 0.18 mM Ca(NO_3_)_2_; 0.12 mM MgSO_4_·7H_2_O; 1.5 mM HEPES; at pH 7.4) with a final DMSO concentration of 1%. Test solutions were freshly prepared for each experiment and were renewed every 24 h. The maximum tested compound concentration was 100 µM and four additional concentrations were also prepared by applying a 1:2 dilution factor. A 1% DMSO solution was used as a vehicle control.

Six-well plates were incubated at 26 ± 1 °C under a 14:10 light:dark cycle for 72 h. To assess the potential lethality of the new compounds, we followed, every 24 h, the four parameters that are outlined in the OECD Guideline No. 236: embryo coagulation, lack of somite formation, non-detachment of the tail, and absence of a heartbeat (from 48 h onward). The percentage of lethality at each concentration that was tested for the new compounds was recorded and expressed as mean ± SEM from three or more independent experiments. The lowest observed adverse effect concentration (LOAEC), which is the lowest concentration causing significant lethality, and the lethal concentration for 50% mortality (LC_50_) were calculated using GraphPad Prism^®^ software v10. LOAEC values were determined through an ANOVA test with Bonferroni’s post-hoc test for multiple comparisons (significance threshold: *p* < 0.05). LC_50_ values were calculated by fitting the mean results from all tested groups in three or more experiments per compound to a non-linear concentration–response curve with variable slope and least squares fit, fixing the minimum and maximum values at 0% and 100% lethality.

## 3. Results and Discussion

### 3.1. Synthesis of the Target Hybrids ***4***–***7***

Compounds **4**–**7** were synthesized through a three-step sequence, which involved the initial alkylation of huprine Y [32] with 9-bromononanenitrile, followed by LiAlH_4_ reduction of the resulting nitrile **8**, and final amide coupling of the aminononylhuprine **9** [35] with the corresponding carboxylic acid (1.1 equiv.), either anthraquinone-2-carboxylic acid, 9-fluorenone-2-carboxylic acid, 3-benzoylbenzoic acid, or 3-acetylbenzoic acid, using EDC·HCl (1.5 equiv.) and HOBt (1.5 equiv.) as coupling reagents, and Et_3_N as a base (2–2.5 equiv.) (Scheme 1). After silica gel column chromatography purification, the target hybrids **4**–**7** were obtained in 49–70% overall yield from huprine Y.

For chemical and biological characterization purposes, compounds **4**–**7** were converted into their hydrochloride salts by reaction with an Et_2_O solution of HCl.

### 3.2. Biological Activity Profiling of the Target Hybrids ***4***–***7***: In Vitro Inhibition of hAChE, hBChE, hBACE-1, and Aβ42 and Tau Aggregation

Compounds **4**–**7** were designed as structurally simplified analogs of the lead rhein–huprine hybrid **3** to overcome potential size-related limitations in physicochemical and pharmacokinetic properties, while retaining its outstanding multitarget profile, which includes the potent inhibition of multiple biological targets with a key pathogenic role in AD, i.e., AChE, BChE, BACE-1, and Aβ and tau aggregation. Thus, to assess the effects of this structural simplification on both the biological activity and DMPK profiles, we experimentally determined the inhibitory activity of hybrids **4**–**7** on the selected biological targets (this section) as well as their DMPK properties (Section 3.3). Moreover, as a preliminary assessment of the acute toxicity of the simplified hybrids, we evaluated their effects on the survival of zebrafish embryos (Section 3.4).

One of the most prominent pathological features of AD is the occurrence of a marked cholinergic deficit in the central nervous system (CNS), including reduced levels of the neurotransmitter acetylcholine (ACh), which accounts for the cognitive symptoms of the disease and for some early disease mechanisms, such as neuroinflammation [46,47,48]. Because ACh is hydrolyzed by the enzyme AChE, the inhibition of AChE results in increased ACh levels and, hence, in beneficial effects on cognition and neuroinflammation. Indeed, three out of the four globally approved anti-AD drugs (donepezil, rivastigmine, and galantamine) are AChE inhibitors, and most multitarget anti-AD agents under development hit AChE as one of their targets [29]. This is the case for the lead rhein–huprine hybrid **3**, which is a single-digit nanomolar inhibitor of hAChE (IC_50_ = 3.60 nM), and for the new simplified analogs **4**–**7**, which display IC_50_ values in the range of 6–12 nM (Table 3). Thus, the structurally simplified analogs **4**–**7** essentially retain the highly potent hAChE inhibitory activity of the lead **3**.

Together with AChE, another esterase that breaks down the neurotransmitter ACh in the CNS, thereby contributing to AD pathology, is BChE, whose brain activity increases as AD progresses [51,52]. This fact has made selective BChE inhibition or dual AChE/BChE inhibition very interesting attributes in anti-AD drug candidates [53,54,55,56,57,58]. It is well-known that the introduction of a chlorine atom at position 3 of huprines, like in huprine Y, or at the equivalent position 6 of tacrine leads to a higher potency on AChE compared to BChE, and, hence to a selectivity of AChE over BChE [59,60]. Thus, the parent huprine Y was a highly potent hAChE inhibitor (IC_50_ = 1.07 nM) and a moderately potent inhibitor of hBChE (IC_50_ = 181 nM), with an AChE vs. BChE selectivity of 169. Likewise, the lead rhein–huprine hybrid **3** and its simplified analogs **4** and **5** were selective for hAChE inhibition, with selectivities very similar to that of huprine Y, i.e., 172, 181, and 149, respectively (Table 3). Strikingly, the most simplified hybrids **6** and **7** were very potent inhibitors of both hAChE (IC_50_ = 11.6 nM and 5.97 nM, respectively) and hBChE (IC_50_ = 16.5 nM and 12.7 nM, respectively). Thus, the structural simplification from the dihydroxyanthraquinone hybrid **3** to the benzophenone and acetophenone derivatives **6** and **7** led to a 40–50-fold increase in hBChE inhibitory activity, with these compounds displaying an interesting dual inhibition profile of these two important AD targets.

Apart from the cholinergic deficit, the extra- or intra-neuronal aggregation of the amyloidogenic Aβ peptides and tau protein, respectively, are the most important hallmarks and key underlying mechanisms of AD [61,62,63,64,65]. Aβ peptides, among which the form with 42 amino acids (Aβ42) is the most aggregation-prone and neurotoxic, are generated by proteolytic cleavage from the amyloid precursor protein (APP). The first and rate-limiting step of APP cleavage is catalyzed by the enzyme BACE-1, which is another prime target in AD drug discovery [66,67]. The lead rhein–huprine hybrid **3**, but not the parent huprine Y, was found to inhibit BACE-1 [35]. Like AChE, BACE-1 has a large active site groove where the relatively large molecules of linked hybrids, like **3**, may be well accommodated [38]. In this case, the huprine moiety of the hybrid **3**, protonated at physiological pH, may interact with the aspartate dyad of BACE-1 at the catalytic site, whereas the rhein moiety may interact with a secondary enzyme binding site through the H-bonding of the carbonyl group of the most hydrophobic edge (position 9) of the dihydroxyanthraquinone ring system with the guanidinium group of Arg307 [36]. These interactions were expected to be conserved in the simplified analogs **4**–**7**, as they keep the huprine moiety and, as an H-bond acceptor for the interaction with Arg307, the carbonyl group of an anthraquinone, fluorenone, benzophenone or acetophenone moiety. Indeed, hybrids **4**–**7** turned out to be active against hBACE-1, especially those bearing a tricyclic system like the lead **3**, i.e., the anthraquinone analog **4** and the fluorenone derivative **5**. Hybrids **4** and **5** essentially retained the single-digit micromolar inhibitory potency of the lead **3** and are equipotent to the reference BACE-1 inhibitor myricetin (Table 3), whereas the most simplified analogs **6** and **7** still have moderate BACE-1 inhibitory activity, with IC_50_ values around or below 15 µM.

Molecules that contain two extended π-systems connected through a tether chain of suitable length have been found to inhibit the aggregation of amyloidogenic proteins, including Aβ and tau [38,50]. This is the case for the lead rhein–huprine hybrid **3**, which inhibited the aggregation of Aβ42 and tau by 74.2% and 58.2%, respectively, when used at a 10 µM concentration in a culture of *E. coli* cells that overexpressed either Aβ42 or tau. Of note, this is a simplified in vivo assay in which the compounds must be internalized in live *E. coli* cells before eliciting their effects on the aggregation of these amyloidogenic proteins [68]. Thus, the positive outcomes obtained in this assay do not only result from an interaction with Aβ42 or tau but also from a favorable membrane permeability. Gratifyingly, the simplified analogs **4**–**6** retained the potency of the lead **3** against Aβ42 aggregation, with percentages of inhibition in the range of 70–73% at 10 µM, and displayed a slightly higher potency than **3** against tau aggregation (62–69% inhibition at 10 µM), comparing well with the reference anti-aggregating compound DP128 [50] (Table 3). Interestingly, the most simplified analog **7**, despite bearing a less extended π-system (acetophenone) relative to the anthraquinone, fluorenone, and benzophenone moieties of the analogs **4**–**6**, was one of the most potent Aβ42 and tau anti-aggregating hybrids.

### 3.3. In Vitro DMPK Property Profiling of the Target Hybrids ***4***–***7***: Aqueous Solubility, Plasma Stability, and Brain Permeability

Once we confirmed that the simplified analogs **4**–**7** essentially retained or even improved the potency of the lead **3** on its multiple targets, we assessed the effects of the structural simplification on some physicochemical and DMPK properties, namely aqueous solubility, plasma stability, and the ability the pass from plasma to the brain, where the biological targets are located. 

Aqueous solubility is a necessary attribute of drugs, since it plays a crucial role in essentially all stages of drug pharmacokinetics, from gastrointestinal absorption to distribution to organs and tissues, and excretion. For drug discovery purposes, kinetic solubility is usually measured. For this assay, drug candidates are dissolved in DMSO and then an aliquot of that solution is diluted in an aqueous buffer, such as PBS, and incubated at 37 °C for several hours. This way, kinetic solubility methods mimic the conditions in which most biological profiling assays are performed and abolish potential solubility variance among different solid forms of the target compounds, while requiring very small amounts of samples and short experiment times [69]. Thus, the kinetic aqueous solubility of the new compounds **4**–**7** and the lead **3** was determined by a nephelometric method, measuring their precipitation after serial dilutions in DMSO (1%)/PBS (99%) and incubation at 37 °C for 2 h [70]. Under these conditions, the aqueous solubility of the lead **3** was 8.6 µM, very similar to that of the other two analogs containing the tricyclic system of anthraquinone (**4**: 8.4 µM) and fluorenone (**5**: 8.1 µM), while the solubility of the ring-opened benzophenone analog **6** was slightly higher (12.5 µM) (Table 4). In all these cases, the solubilities were below the value of 10 µg/mL (6.3, 5.9, 5.4, and 8.4 µg/mL, respectively), so they can be classified as sparingly soluble (<10 µg/mL) [70]. Gratifyingly, the solubility of the most simplified analog, the acetophenone analog **7**, was doubled compared to the lead **3**, reaching a value of 18.1 µM (11.0 µg/mL), and hence the range of partially soluble (10–100 µg/mL). Even though greater aqueous solubility would be desirable for all these compounds, they were soluble at concentrations that are higher or similar to those at which they are active against their multiple targets, especially in the case of the most simplified analog **7**, which is in agreement with the expected improvement of physicochemical properties upon structural simplification.

Hybrids **3**–**7** contain an amide group at one of the ends of the linker that connects the two constituting pharmacophoric moieties, which might be amenable to hydrolysis by plasma hydrolytic enzymes. This would lead to rapid clearance and, hence, poor brain penetration and low efficacy. The stability of the novel compounds and the lead **3** in plasma of three species (human, mouse, and rat) at 37 °C was assessed at different times. All the compounds were found to be very stable, with essentially no hydrolysis after 1 h of incubation (Table 4). After 6 h of incubation with human and rat plasma, the lead **3** was partially hydrolyzed (15.6% and 32.1% hydrolyzed, respectively), whereas it remained still quite stable in mouse plasma, with only 7.1% being hydrolyzed. The stability after 6 h of incubation of the new hybrid **5** was similar to that of the lead **3**, while hybrids **4**, **6**, and **7** were, in general, more stable than the lead **3**. The most stable compound turned out to be the most simplified analog **7**, which remained essentially unchanged after 6 h of incubation with human and mouse plasma, with only 14.2% hydrolysis in rat plasma (Table 4).

Once we confirmed that the lead **3** and the new analogs **4**–**7** had an acceptable aqueous solubility and high stability up to 6 h in plasma, we assessed their ability to cross from plasma to the brain, where these compounds are expected to elicit their multiple actions. For this purpose, we employed the well-established PAMPA-BBB assay [41]. After assay validation with fourteen known drugs, the threshold for high BBB permeation (CNS+) was established at values of permeability (*P*_e_) over 5.21 × 10^−6^ cm/s. Like the lead **3**, all the simplified analogues **4**–**7** displayed favorable BBB permeabilities, clearly over the CNS+ threshold (Table 4).

Overall, all these assays confirmed a favorable DMPK property profile of the new hybrids, especially in the case of the most simplified hybrid **7**, which displayed the highest BBB permeability among all the new hybrids, as well as greater aqueous solubility and plasma stability than the lead **3**.

### 3.4. Acute Toxicity of the Target Hybrids ***4***–***7*** in Zebrafish Embryos

**Figure 2:**
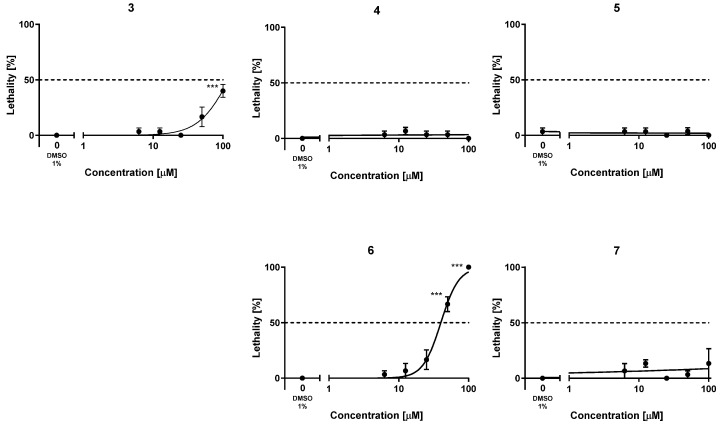
Lethality–concentration curves. Plotted results were obtained upon exposure of zebrafish embryos to five increasing concentrations of the new hybrids **4**–**7** and the lead **3** in the range of 1–100 µM. The results are shown as the mean ± SEM of at least three independent experiments, using ten embryos for each concentration in each experiment, i.e., using a total of thirty embryos per concentration; *** *p* < 0.001 by ANOVA and Bonferroni’s post-hoc test for multiple comparisons.

To assess the safety profile of the new hybrids **4**–**7**, their acute toxicity was evaluated using the fish embryo acute toxicity (FET) test based on the OECD Test Guideline 236 [38]. For comparison purposes, the acute toxicity of the lead **3** was also evaluated for the first time. This assay is being increasingly used in early preclinical studies of new drug candidates, partially replacing studies with rodents, leveraging a reduced requirement of number of animals, costs, and time [71,72]. The lethality of these compounds was assessed by exposing fertilized, synchronously divided zebrafish embryos to increasing concentrations of the compounds, from 1 to 100 µM, in a solution of Danieau’s medium containing 1% DMSO, every 24 h, finalizing the experiments at 72 h. Every 24 h, the lethality of the compounds was assessed in terms of coagulation of the embryos, lack of somite formation or tail bud detachment, and, from 48 h on, the lack of heartbeat was also monitored. After 72 h, the lowest concentration of each compound that caused significant adverse effects for lethality (LOAEC) and the concentration that killed 50% of individuals (LC_50_) were calculated. The lead compound **3** turned out to be quite safe in this assay, with an LC_50_ value above (but close to) 100 µM (Table 5, Figure 2). Indeed, at 100 µM, the lead **3** elicited significant adverse effects (LOAEC = 100 µM). With the sole exception of hybrid **6**, which had a higher acute toxicity than the lead **3** (LC_50_ = 40.1 µM), the simplified analogs were less toxic than the lead compound. With both LOAEC and LC_50_ values apparently much higher than 100 µM (Table 5, Figure 2), the acute toxicity of the new analogs **4**, **5**, and **7** appeared to be at concentrations that are several orders of magnitude higher than those at which they are active against their multiple targets, thereby confirming a favorable safety profile.

## 4. Conclusions

Poor physicochemical and PK properties are the Achilles’ heel of many linked hybrids, which hampers their preclinical and clinical development despite the huge therapeutic potential that may result from their multitarget profile. Here we have shown that the simplification of the chemical structure of a large-sized anti-Alzheimer rhein–huprine hybrid lead can be done while retaining or even surpassing the biological activities of the lead and improving the DMPK properties and even the safety profile, and hence the greater likelihood of the simplified analogs becoming a drug. Indeed, the most simplified analog, the acetophenone derivative **7**, exhibited a very interesting activity–property–safety profile encompassing (i) activity against multiple prime biological targets for AD treatment, namely the dual highly potent inhibition of hAChE and hBChE, in addition to a moderately potent inhibitory activity against hBACE-1 and Aβ42 and tau aggregation; (ii) better DMPK properties than the initial lead, including a 2-fold increased aqueous solubility, higher plasma stability, and favorable BBB permeability; and (iii) lower acute toxicity in a model organism such as zebrafish embryos, thereby emerging as a promising optimized lead for AD drug discovery.

## Figures and Tables

**Table 1:** Gradient used for compound quantification in the plasma stability assay.

Time (min)	Water	Acetonitrile
0	95%	5%
0.1	95%	5%
1	0%	100%
2	0%	100%
2.1	95%	5%
2.5	95%	5%

**Table 2:** Described and experimental PAMPA-BBB permeability (*P*e, 10^−6^ cm s^−1^) values of the drugs used for assay validation.

Drug	Literature *P*e Value ^1^	Experimental *P*e Value
Cimetidine	0.0	0.7 ± 0.0
Norfloxacin	0.1	0.9 ± 0.0
Ofloxacin	0.8	1.1 ± 0.1
Lomefloxacin	1.1	0.8 ± 0.1
Hydrocortisone	1.9	1.4 ± 0.0
Piroxicam	2.5	2.2 ± 0.1
Clonidine	5.3	6.5 ± 0.0
Corticosterone	5.1	6.7 ± 0.1
Promazine	8.8	13.8 ± 0.3
Progesterone	9.3	16.8 ± 0.0
Desipramine	12.0	17.8 ± 0.1
Imipramine	13.0	12.3 ± 0.1
Verapamil	16.0	28.1 ± 0.8
Testosterone	17.0	26.5 ± 0.7

^1^ Literature *P*e values reported in [41].

**Table 3:** In vitro inhibitory activity of the new compounds **4**–**7**, the lead rhein–huprine hybrid **3**, and reference compounds on hAChE, hBChE, hBACE-1, and Aβ42 and tau aggregation.

Compd.	hAChE IC_50_ (nM) ^1^	hBChE IC_50_ (nM) ^1^	hBACE-1 IC_50_ (µM) or (% inh. @15 µM) ^2^	Aβ42 Aggregation ^3^ % inh. @10 µM	Tau Aggregation ^3^ % inh. @10 µM
**3**	3.60 ± 0.21	620 ± 20	1.50 ± 0.38	74.2 ± 1.6	58.2 ± 1.8
**4**	6.91 ± 0.77	1250 ± 130	2.89 ± 0.50	73.3 ± 3.6	63.0 ± 3.5
**5**	5.93 ± 0.26	883 ± 55	5.63 ± 0.01	69.8 ± 1.2	68.9 ± 2.1
**6**	11.6 ± 0.9	16.5 ± 1.9	(61.5 ± 0.4)	72.6 ± 2.7	62.5 ± 2.1
**7**	5.97 ± 0.93	12.7 ± 0.9	(48.1 ± 0.3)	73.1 ± 4.8	68.3 ± 3.1
**HupY** ^4^	1.07 ± 0.05	181 ± 15			
**Myr** ^5^			4.18 ± 0.50		
**DP128** ^6^				78.1 ± 1.7	69.9 ± 0.7

^1^ Compound concentration that produces 50% inhibition of the activity of human recombinant AChE or human serum BChE. IC_50_ values are shown as the mean ± standard error of at least two independent experiments performed in triplicate. ^2^ Compound concentration that produces 50% inhibition of the activity of human recombinant BACE-1 or percentage inhibition of the activity of human recombinant BACE-1 produced by the compound at a 15 µM concentration; in the latter case, % inhibition values are parenthesized. Activity values are indicated as the mean ± standard deviation of the mean of two independent experiments performed in triplicate. ^3^ Percentage inhibition of the Aβ42 and tau aggregation produced by the compound at a 10 µM concentration in live *E. coli* cells that overexpressed these amyloidogenic proteins. Activity values are indicated as mean ± standard error of the mean of nine independent experiments performed in triplicate. ^4^ HupY = huprine Y, used as a reference AChE and BChE inhibitor [32]. ^5^ Myr = myricetin, used as a reference BACE-1 inhibitor [49]. ^6^ DP128, used as a reference Aβ42 and tau aggregation inhibitor [50].

**Table 4:** Aqueous solubility, plasma stability, and in vitro brain permeability of the new compounds **4**–**7** and the lead rhein–huprine hybrid **3**.

Compd.	Solubility ^1^ (µM)	Plasma Stability ^2^ % Remaining Compound (h/m/r)		Brain Permeability ^3^ *P*_e_ (10^−6^ cm s^−1^)
		1 h	6 h	
**3**	8.6	100/97.2/98.0	84.4/92.9/67.9	21.5 ± 0.7 (CNS+)
**4**	8.4	94.2/96.0/100	97.6/90.1/77.2	9.2 ± 1.2 (CNS+)
**5**	8.1	88.5/95.7/93.6	80.4/90.4/84.7	6.2 ± 0.8 (CNS+)
**6**	12.5	100/98.3/96.0	90.8/76.9/92.4	7.4 ± 0.3 (CNS+)
**7**	18.1	100/97.5/98.9	100/96.0/85.8	16.5 ± 1.1 (CNS+)

^1^ Experimentally determined kinetic solubility of the target compounds at 37 °C in PBS with 1% DMSO. For assay validation, the following compounds were used: progesterone (described, 3.7 µM [36]; found, 5.9 µM), digoxin (described, 59.3 µM [36]; found, 86.3 µM). ^2^ Percentage of remaining compound in human (h), mouse (m), and rat (r) plasma after 1 h and 6 h of incubation at 37 °C. ^3^ Experimental permeability (*P*_e_) determined in the PAMPA-BBB assay. The reference compounds used in assay validation are shown in Table 2. High brain permeability (CNS+) when *P*_e_ (10^−6^ cm s^−1^) > 5.2; low brain permeability (CNS−) when *P*_e_ (10^−6^ cm s^−1^) < 1.9; uncertain brain permeability when 5.2 > *P*_e_ (10^−6^ cm s^−1^) > 1.9.

**Table 5:** Acute toxicity of the new compounds **4**–**7** and the lead rhein–huprine hybrid **3** in zebrafish embryos after 72 h of exposure.

Compd.	LOAEC ^1^ (µM)	LC_50_^2^ (µM; CI_95_)
**3**	100	>100
**4**	>100	>100
**5**	>100	>100
**6**	50	40.1 (35.4–45.2)
**7**	>100	>100

^1^ LOAEC, or the lowest observed adverse effect concentration, is the minimum concentration of a compound that results in significant adverse effects for lethality. The calculation was performed using an ANOVA test followed by Bonferroni’s post-hoc test for multiple comparisons (significance level, *p* < 0.05). ^2^ LC_50_, or the lethal concentration for 50% mortality, represents the concentration of a compound killing 50% of individuals. It was determined by fitting the mean results from three or more experiments per compound to a non-linear concentration–response curve with a variable slope and least squares fit, with lethality fixed at a minimum of 0% and a maximum of 100%. LOAEC and LC_50_ values were derived from three or more independent experiments, each involving ten embryos per concentration, five different concentrations of each compound, and a solvent control group (1% DMSO).

## Data Availability

The original contributions presented in the study are included in the article and Supplementary Material.

## Supplementary Materials

The following supporting information can be downloaded at: https://www.mdpi.com/article/10.3390/pharmaceutics16080982/s1, Figure S1: ^1^H (400 MHz, CD_3_OD) and ^13^C (100.6 MHz, CD_3_OD) NMR spectra and HPLC traces of compounds **4**–**7**.
