# Supplementary material for: Stepwise Structural Simplification of the Dihydroxyanthraquinone Moiety of a Multitarget Rhein-Based Anti-Alzheimer Lead to Improve Drug Metabolism and Pharmacokinetic Properties
Source: Pharmaceutics. 2024 Jul 25;16(8):982. doi: 10.3390/pharmaceutics16080982 (PMC11359831; doi:10.3390/pharmaceutics16080982)
Supplement: Supplementary file 1 [file pharmaceutics-16-00982-s001.zip › pharmaceutics-3101614-supplementary.pdf]

Supplementary Materials

Stepwise Structural Simplification of the Dihydroxyanthraquinone Moiety of a Multitarget Rhein-Based Anti-Alzheimer Lead to Improve Drug Metabolism and Pharmacokinetic Properties

Caterina Pont [1], Anna Sampietro [1,2], F. Javier Pérez-Areales [1,2], Nunzia Cristiano [1], Agustí Albalat [1], Belén Pérez [3], Manuela Bartolini [4], Angela De Simone [5], Vincenza Andrisano [6], Marta Barenys [7,8], Elisabet Teixidó [7,9], Raimon Sabaté [10], M. Isabel Loza [11], José Brea [11] and Diego Muñoz-Torrero [1,2*]

[1] Laboratory of Medicinal Chemistry (CSIC Associated Unit), Faculty of Pharmacy and Food Sciences, University of Barcelona, Av. Joan XXIII 27-31, E-08028 Barcelona, Spain; aitak1989@gmail.com (C.P.); annasampietro@ub.edu (A.S.); fjperezareales@ub.edu (F.J.P.-A.); nunzia.cristiano1991@gmail.com (N.C.); agusalbalat@gmail.com (A.A.)

[2] Institute of Biomedicine of the University of Barcelona (IBUB), E-08028 Barcelona, Spain

[3] Department of Pharmacology, Therapeutics and Toxicology, Autonomous University of Barcelona, E-08193 Bellaterra, Spain; belen.perez@uab.cat

[4] Department of Pharmacy and Biotechnology, University of Bologna, Via Belmeloro, 6, I-40126 Bologna, Italy; manuela.bartolini3@unibo.it

[5] Department of Drug Science and Technology, University of Turin, I-10125 Torino, Italy; angela.desimone@unito.it

[6] Department for Life Quality Studies, Alma Mater Studiorum University of Bologna, Corso d'Augusto 237, I-47921 Rimini, Italy; vincenza.andrisano@unibo.it

[7] Toxicology Unit, Department of Pharmacology, Toxicology and Therapeutic Chemistry, Faculty of Pharmacy and Food Sciences, University of Barcelona, Av. Joan XXIII 27-31, E-08028 Barcelona, Spain; mbarenys@ub.edu (M.B.); eteixido1511@ub.edu (E.T.)

[8] German Centre for the Protection of Laboratory Animals (Bf3R), German Federal Institute for Risk Assessment (BfR), 10589 Berlin, Germany

[9] Institute of Nutrition and Food Safety of the University of Barcelona (INSA-UB), E-08921 Santa Coloma de Gramenet, Spain

[10] Department of Pharmacy and Pharmaceutical Technology and Physical-Chemistry, Faculty of Pharmacy and Food Sciences, University of Barcelona, Av. Joan XXIII 27-31, E-08028 Barcelona, Spain; rsabate@ub.edu

[11] BioFarma Research Group, Centro Singular de Investigación en Medicina Molecular y Enfermedades Crónicas (CIMUS), Departamento de Farmacología, Farmacia y Tecnología Farmacéutica, Universidade de Santiago de Compostela, Av. de Barcelona s/n, E-15782, Santiago de Compostela, Spain; mabel.loza@usc.es (M.I.L.); pepo.brea@usc.es (J.B.)

* Correspondence: dmunoztorrero@ub.edu

Content: ^{1}H (400 MHz, CD_3OD) and ^{13}C (100.6 MHz, CD_3OD) NMR Spectra and HPLC traces of compounds 4-7

N-{9-[(3-Chloro-6,7,10,11-tetrahydro-9-methyl-7,11-methanocycloocta[*b*]quinolin-12-yl)amino]nonyl}anthraquinone-2-carboxamide (**4**)

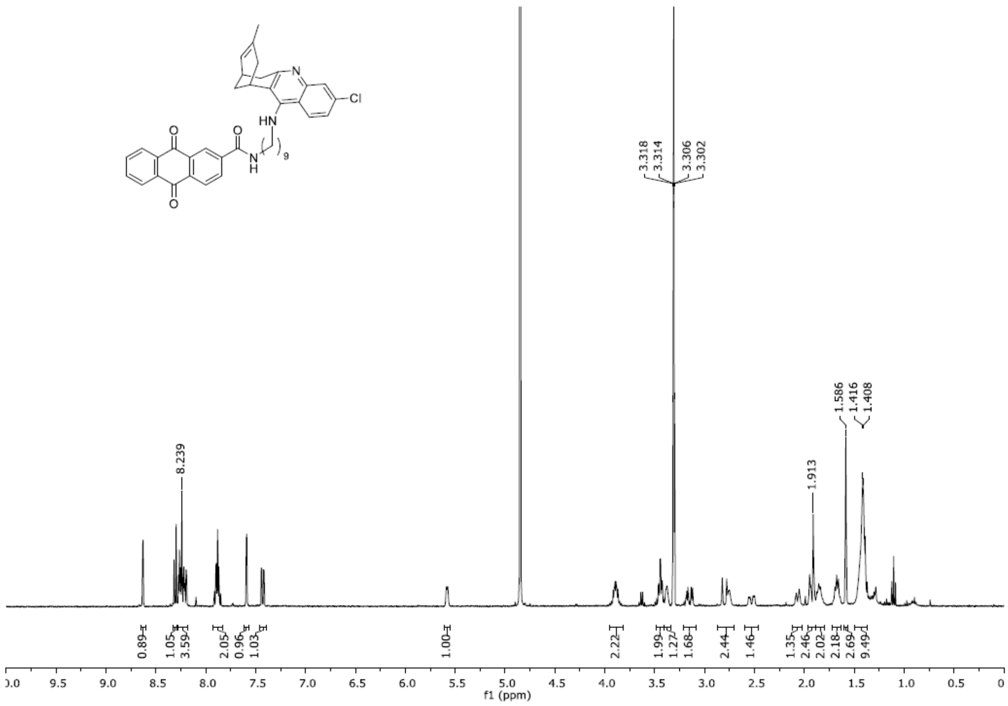

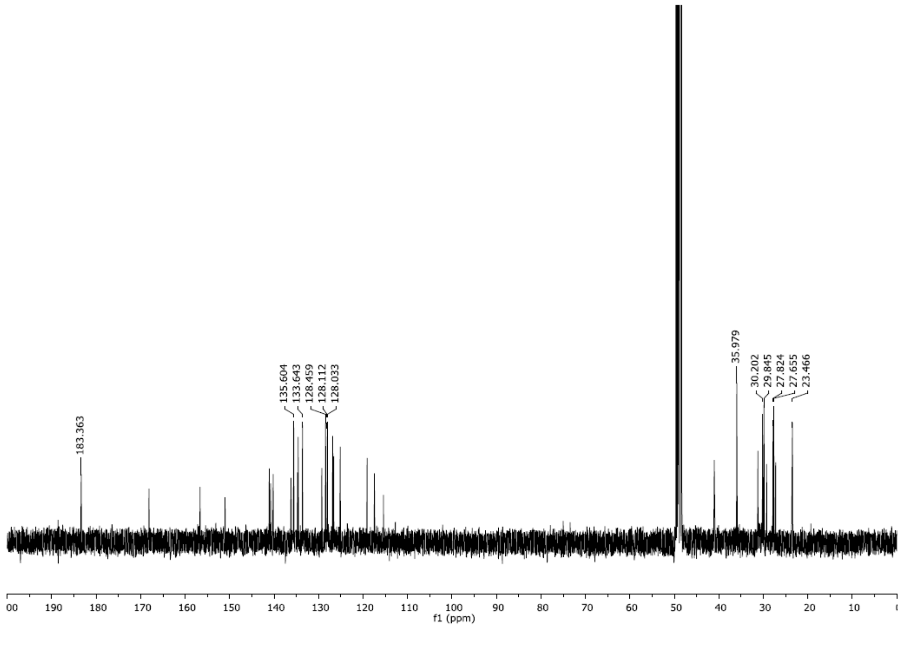

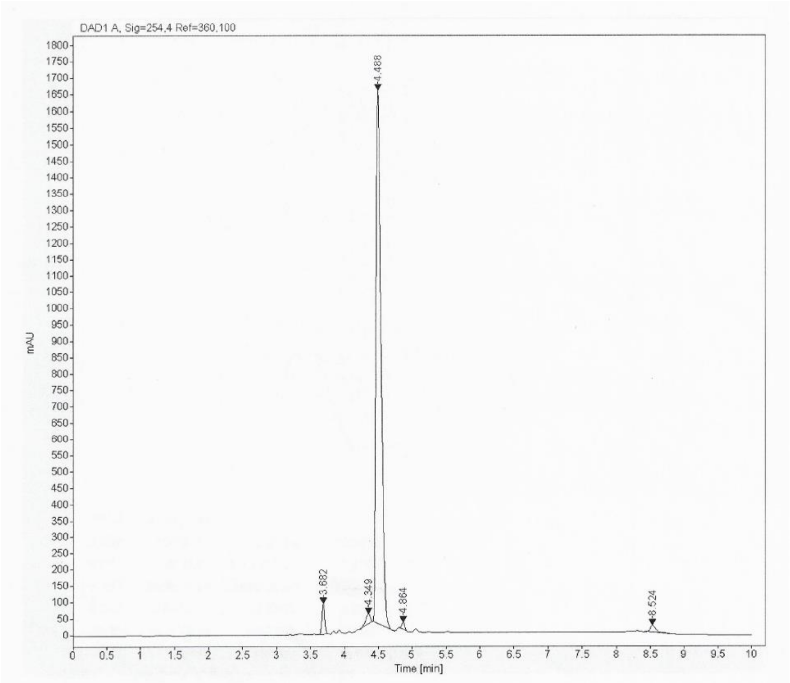

Signal: DAD1 A, Sig=254,4 Ref=360,100

RT [min]	Area	Height	Area%	Name
3.682	272.3674	93.6207	2.9543	
4.349	156.2413	31.3123	1.6947	
4.488	8596.1016	1633.1886	93.2412	
4.864	66.4281	22.3577	0.7205	
8.524	128.0745	22.5968	1.3892	
Sum	9219.2130			

N-{9-[(3-Chloro-6,7,10,11-tetrahydro-9-methyl-7,11-methanocycloocta[*b*]quinolin-12-yl)amino]nonyl}-9-fluorenone-2-carboxamide (**5**)

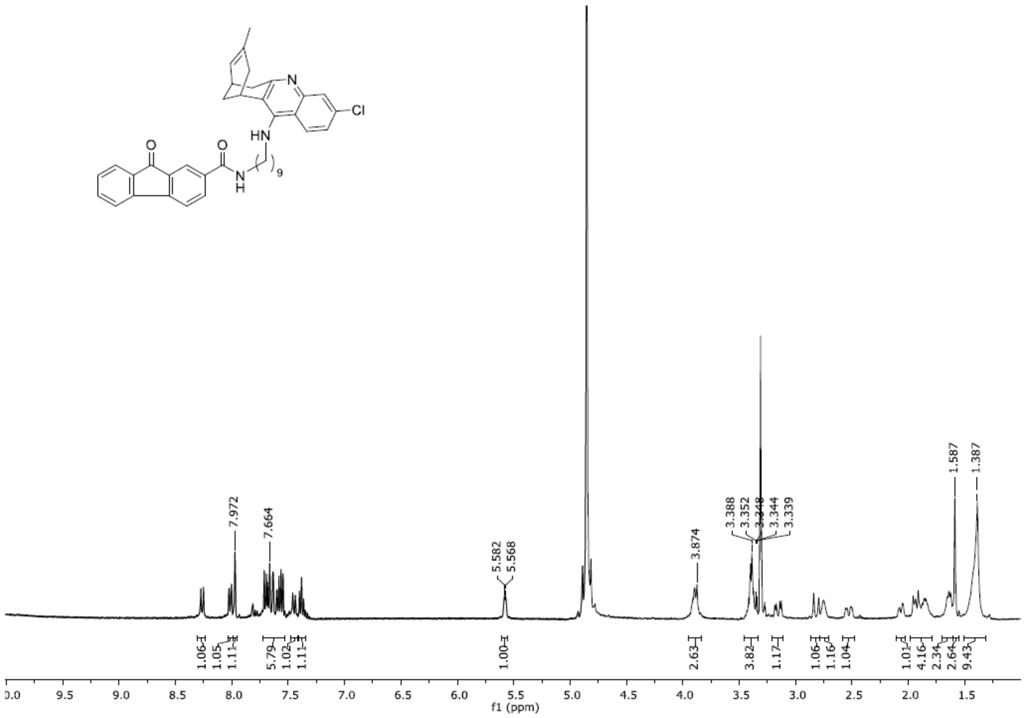

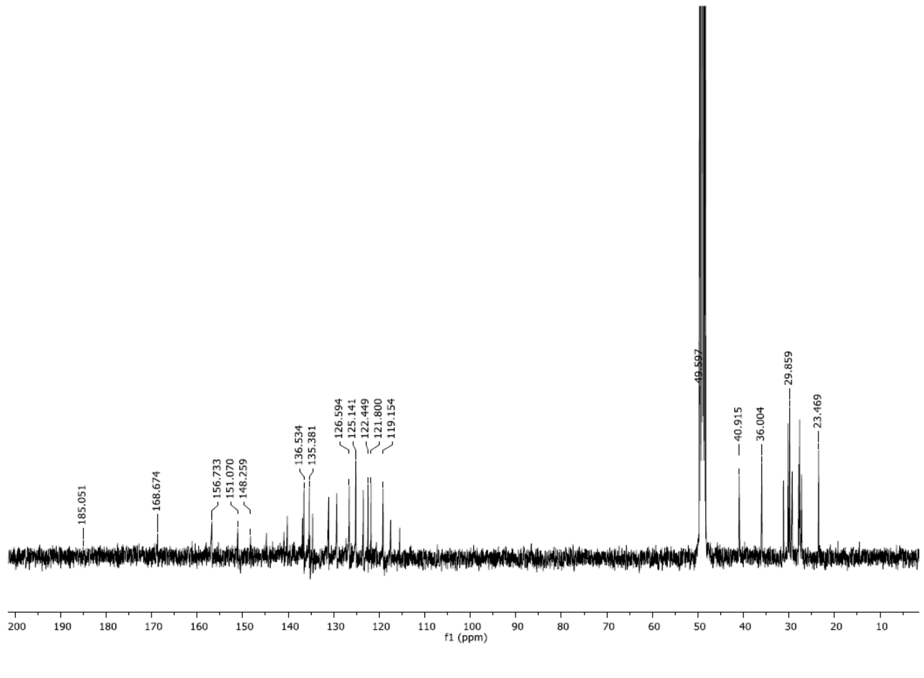

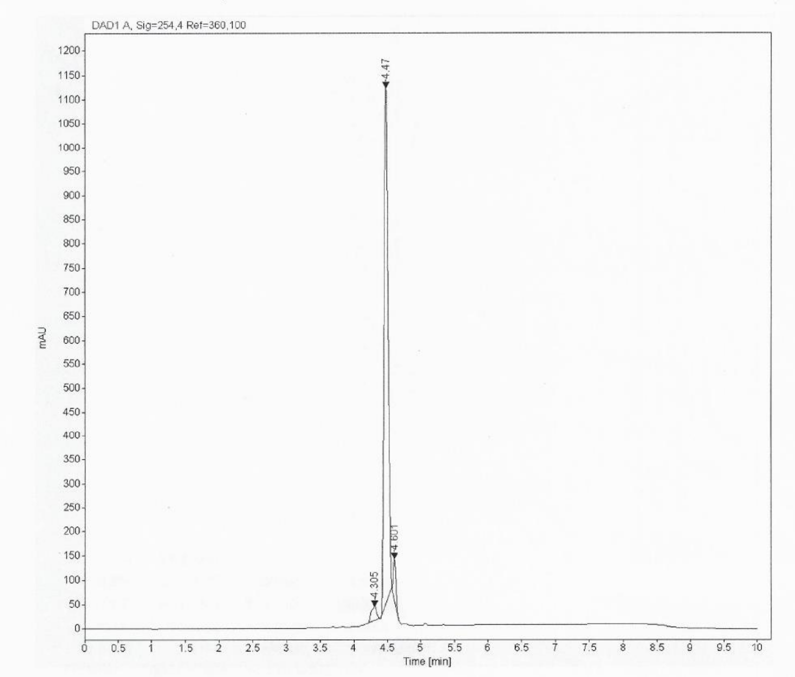

Signal: DAD1 A, Sig=254,4 Ref=360,100

RT [min]	Area	Height	Area%	Name
4.305	167.2553	29.8949	3.3676	
4.470	4581.6655	1084.4165	92.2508	
4.601	217.6132	82.4882	4.3816	
Sum	4966.5341			

3-Benzoyl-*N*-{9-[(3-chloro-6,7,10,11-tetrahydro-9-methyl-7,11-methanocycloocta[*b*]quinolin-12-yl)amino]nonyl}benzamide (**6**)

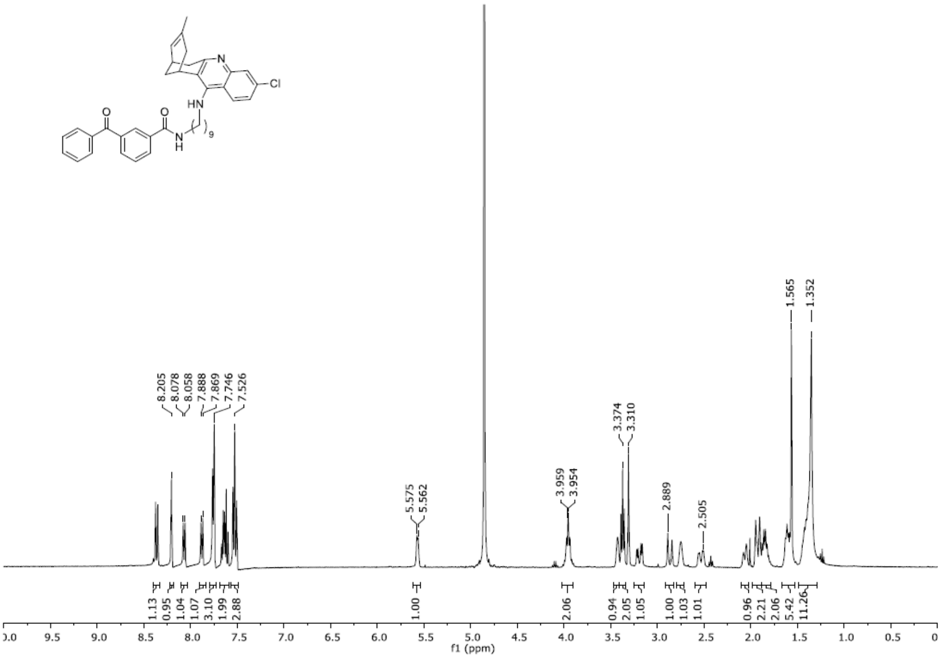

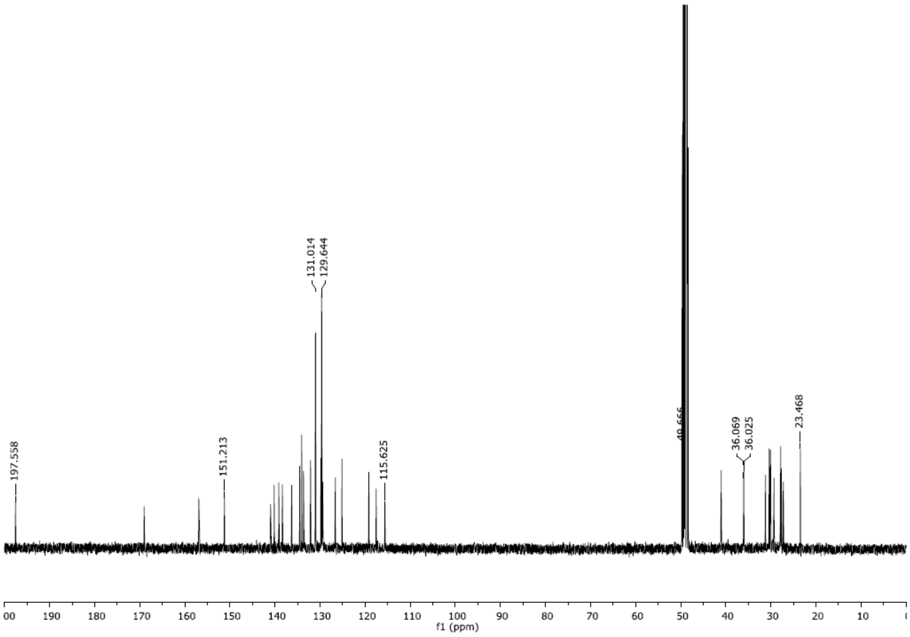

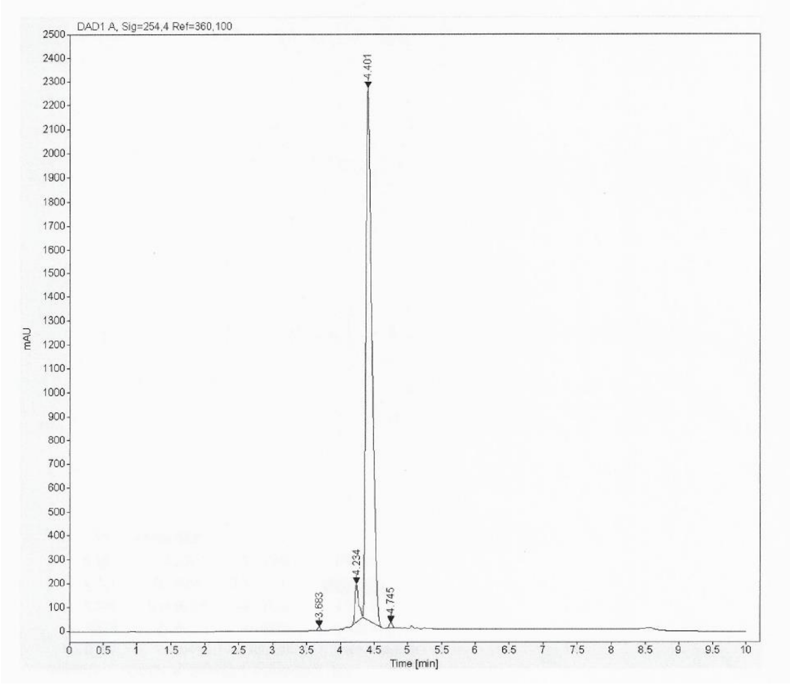

Signal: DAD1 A, Sig=254,4 Ref=360,100

RT [min]	Area	Height	Area%	Name
3.683	47.5711	17.0276	0.3243	
4.234	619.9299	163.4215	4.2261	
4.401	13921.6680	2227.1240	94.9060	
4.745	79.7383	25.4908	0.5436	
Sum	14668.9072			

3-Acetyl-*N*-{9-[(3-chloro-6,7,10,11-tetrahydro-9-methyl-7,11-methanocycloocta[*b*]quinolin-12-yl)amino]nonyl}benzamide (**7**)

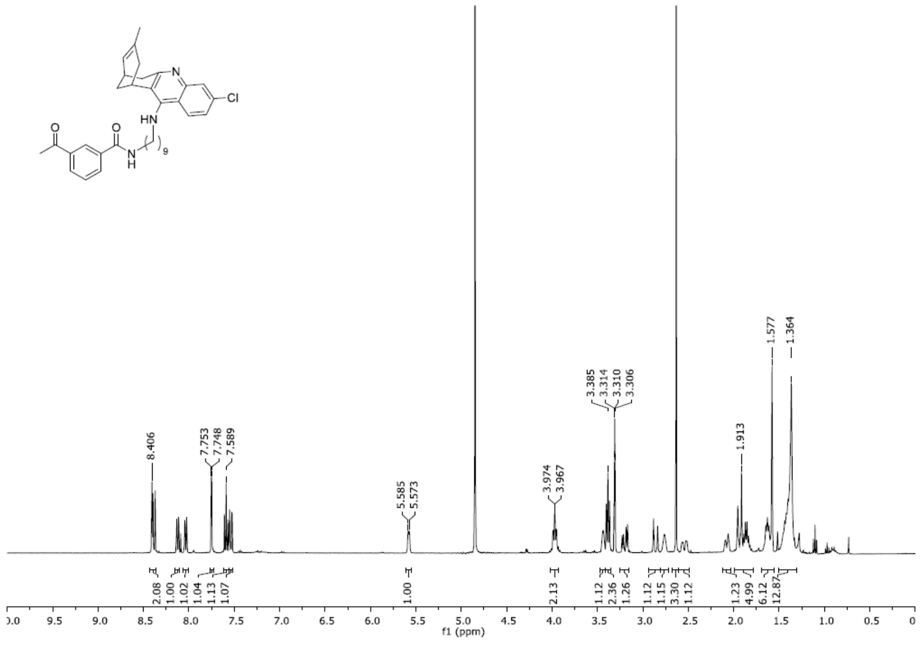

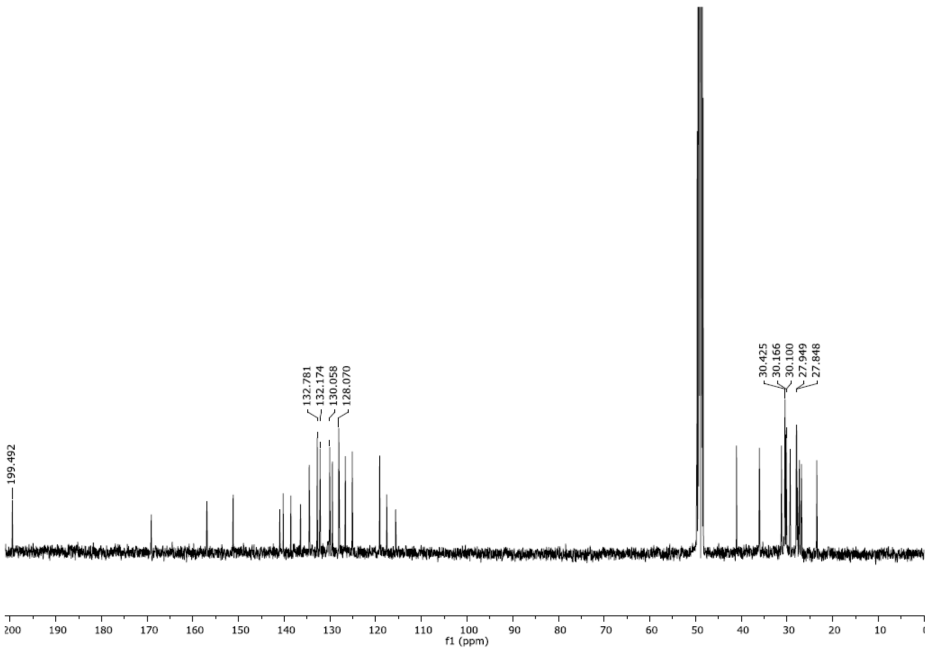

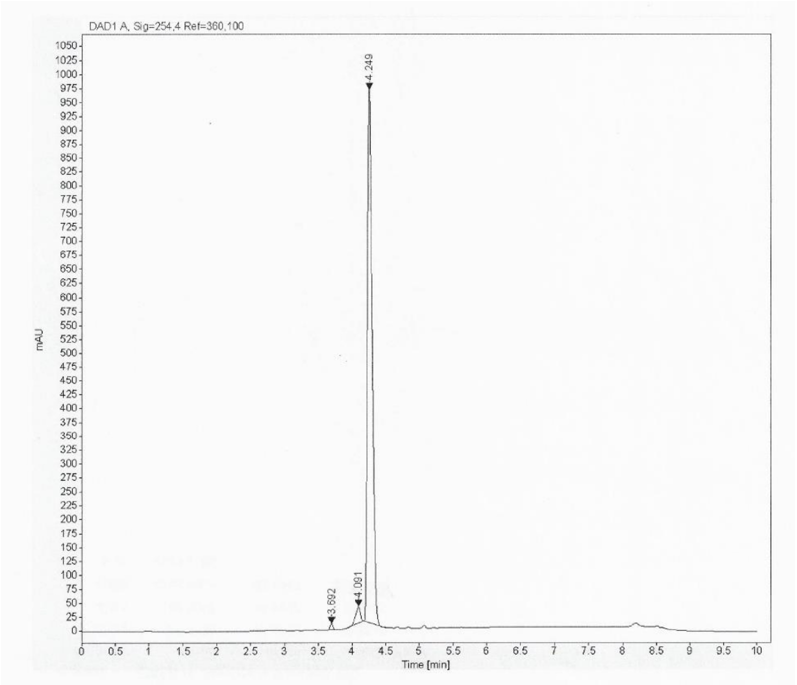

Signal: DAD1 A, Sig=254,4 Ref=360,100

RT [min]	Area	Height	Area%	Name
3.692	37.5779	12.5675	0.7545	
4.091	149.0363	30.9493	2.9925	
4.249	4793.6826	963.8442	96.2529	
Sum	4980.2969			
